# Interfering with lipid metabolism through targeting CES1 sensitizes hepatocellular carcinoma for chemotherapy

**DOI:** 10.1172/jci.insight.163624

**Published:** 2023-01-24

**Authors:** Gang Li, Xin Li, Iqbal Mahmud, Jazmin Ysaguirre, Baharan Fekry, Shuyue Wang, Bo Wei, Kristin L. Eckel-Mahan, Philip L. Lorenzi, Richard Lehner, Kai Sun

**Affiliations:** 1 Center for Metabolic and Degenerative Diseases, The Brown Foundation Institute of Molecular Medicine for the Prevention of Human Diseases, University of Texas Health Science Center at Houston, Houston, Texas, USA.; 2 Metabolomic Core Facility, Department of Bioinformatics and Computational Biology, The University of Texas MD Anderson Cancer Center, Houston, Texas, USA.; 3 Department of Integrative Biology and Pharmacology, The University of Texas Health Science Center at Houston, Houston, Texas, USA.; 4 Program in Biochemistry and Cell Biology, MD Anderson Cancer Center-UTHealth Graduate School of Biomedical Sciences, Houston, Texas, USA.; 5 Group on Molecular and Cell Biology of Lipids, Department of Pediatrics, University of Alberta, Alberta, Canada.

**Keywords:** Metabolism, Fatty acid oxidation, Signal transduction

## Abstract

Hepatocellular carcinoma (HCC) is the most common lethal form of liver cancer. Apart from surgical removal and transplantation, other treatments have not yet been well established for patients with HCC. In this study, we found that carboxylesterase 1 (CES1) is expressed at various levels in HCC. We further revealed that blockage of CES1 by pharmacological and genetical approaches leads to altered lipid profiles that are directly linked to impaired mitochondrial function. Mechanistically, lipidomic analyses indicated that lipid signaling molecules, including polyunsaturated fatty acids (PUFAs), which activate PPARα/γ, were dramatically reduced upon CES1 inhibition. As a result, the expression of SCD, a PPARα/γ target gene involved in tumor progression and chemoresistance, was significantly downregulated. Clinical analysis demonstrated a strong correlation between the protein levels of CES1 and SCD in HCC. Interference with lipid signaling by targeting the CES1-PPARα/γ-SCD axis sensitized HCC cells to cisplatin treatment. As a result, the growth of HCC xenograft tumors in NU/J mice was potently slowed by coadministration of cisplatin and CES1 inhibition. Our results, thus, suggest that CES1 is a promising therapeutic target for HCC treatment.

## Graphical abstract

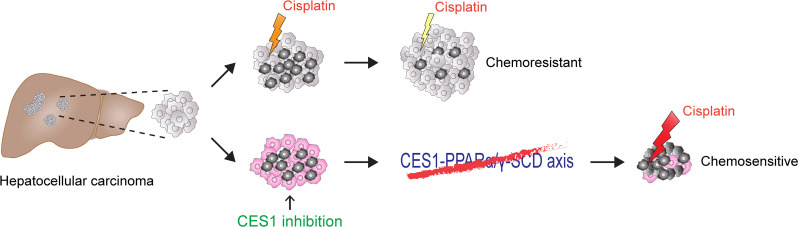

## Introduction

Liver cancer is prevalent worldwide and is ranked as the third leading cause of cancer-related deaths (1). Most adult liver cancers are hepatocellular carcinoma (HCC) and often have a poor prognosis, owing to the lack of effective therapies (2). Currently, the most practical methods for HCC treatment are surgical and transplantation resection. However, the outcomes of surgical approaches are poor, with high recurrence rates (3). Despite many years of dedicated studies, no other standard treatments have been formally established for HCC (4). In this context, cisplatin has recently drawn great clinical attention because of its promising killing effect on advanced HCC. Cisplatin is a chemotherapeutic agent used to treat a wide range of human cancers (5, 6). It exerts an antitumor effect mainly by interfering with genomic DNA replication, which induces DNA damage and apoptosis, thereby killing rapidly proliferating cancer cells. Unfortunately, while initial responsiveness is high, most HCC patients exhibit different degrees of drug insensitivity and chemoresistance upon treatment with cisplatin for prolonged periods (5). Mechanistically, several lipid metabolic pathways have been identified as the key factors that induce cisplatin resistance in HCC (7). Therefore, new combination regimens including cisplatin and interference with lipid metabolism might be an effective strategy to deal with chemoresistance in patients with advanced HCC (5–8).

In addition to hepatitis infections and alcoholic injury, lipid disorder–related liver diseases, such as nonalcoholic fatty liver disease (NAFLD) and nonalcoholic steatohepatitis (NASH), have been directly linked to the development of HCC (9–14). Abnormal lipid metabolism is recognized as a pivotal factor that plays a critical role in HCC development and progression (15). As the primary organ for lipid metabolism, the liver synthesizes fatty acids, which form triglycerides (TG) and other lipids via lipogenesis (16). Different lipid species not only serve as the main energy source via β-oxidation in mitochondria, but also function as key building blocks for the growth of cancer cells. Furthermore, unique free fatty acids (FFAs) produced by lipolysis may act as a “third messenger” to trigger signaling pathways for HCC initiation, progression, and maintenance (17, 18). A fine-tuned balance between lipid biosynthesis, desaturation, and metabolism is key to maintaining normal liver function, and disruptions of this balance can be the cause and consequence of fatty liver diseases and, hence, HCC (19–21). However, limited knowledge of the hepatic lipidome has prevented the development of related therapeutic agents to treat lipid disorder–induced HCC.

The lipid components are assembled into lipid droplets in hepatocytes and other cell types as well. Lipid droplets serve as a major platform for dynamics of lipid metabolism. Many important structural proteins and enzymes, such as perilipins (PLIN1–PLIN5), CIDEA–C, ATGL, and CGI-58, are specifically located on the surface of lipid droplets. They tightly regulate the formation, growth, function, and turnover of the lipid droplets (22). Given their key roles in lipid storage, membrane biosynthesis, lipid signaling, and inflammation in cells, lipid droplets have gradually been recognized as critical organelles in cancer cells (23). Importantly, the components in the lipid droplets of the cancer cells exhibit unique features, including markedly increased levels of monounsaturated fatty acids (MUFA) and polyunsaturated fatty acids (PUFA). These special lipid molecules trigger multiple oncogenic signaling pathways that promote tumor growth (19, 23–25). A key enzyme that converts saturated fatty acids (SFAs) to MUFAs and PUFAs is stearoyl-CoA desaturase 1 (SCD1, referred to as SCD in humans) (26). SCD is ubiquitously expressed in most cancer cells, and its levels are tightly associated with the aggressiveness of cancers (27). Recent studies have highlighted its direct function in cancer cell stemness, proliferation, migration, and metastasis through regulation of lipid signaling pathways and membrane architecture (28–30). More importantly, SCD has also been linked to chemoresistance in certain types of cancers, including HCC (24, 28). Based on previous findings, several specific inhibitors targeting SCD activity have been developed and are under preclinical tests to treat and/or deal with the chemoresistance of certain types of cancers (17, 31). Nevertheless, the lipid signaling–driven pathways that trigger SCD activation in tumor cells remain unclear.

Carboxylesterase 1 (CES1) belongs to a large mammalian serine esterase family (32). CES1 is enriched in metabolically active tissues, including liver and white and brown adipose tissues (33, 34). It catalyzes the hydrolysis of ester and thioester bonds in lipids both in vitro and in vivo and, hence, plays essential roles in lipid metabolism and whole-body energy homeostasis (33). In rodents, its homolog is referred to as Ces1d or Ces3/TGH (32, 33). While it has been considered to exert a catalytic function on lipids in the ER, our recent studies demonstrated that Ces1d directly targets lipid droplets, where it hydrolyzes TG and produces FFAs that promote energy expenditure (34, 35). Particularly, in the liver, CES1 catabolizes lipids and promotes the assembly of apolipoproteins, thereby maintaining whole-body lipid metabolic homeostasis (33, 36, 37). Intriguingly, even though artificially overexpressed CES1 was shown to exert a antiproliferative function in the liver cancer cell line Hep3B, its protein levels have long been considered undetectable in HCC and HCC-derived cell lines, probably because of the lack of high-affinity antibodies that specifically recognize endogenous CES1 (38–40). In this context, the bona fide function and regulation of CES1 in HCC per se remain to be elucidated.

In this study, we used a recently established high-affinity anti-CES1 antibody to analyzed the protein levels of CES1 in an array of human liver tumor samples. The results revealed that the CES1 protein levels were detectable and varied among the samples. We further demonstrated that blockage of CES1 activity by a specific inhibitor WWL229 — which targets the active site of CES1 and, hence, inhibits its enzymatic activity (41) — or by genetic KO led to reprogrammed lipid metabolism. Consequently, mitochondrial function was impaired in response to the inhibition of CES1. Mechanistically, we found that key lipid signaling molecules that potentially trigger PPARα/γ transactivation, including multiple PUFAs, were significantly reduced when the activity of CES1 was blocked. As a result, the expression of SCD, a direct target of PPARα/γ, was dramatically downregulated. The lipid metabolism, whose interference was induced by reduced SCD, potently sensitized HCC cells to chemotherapeutic agents, such as cisplatin treatment. Our findings suggest that CES1 plays a role in regulation of HCC progression and chemoresistance; thus, they pinpoint it as a potential target for HCC therapy.

## Results

### CES1 is selectively expressed at different levels in human liver tumors.

**Figure 1:**
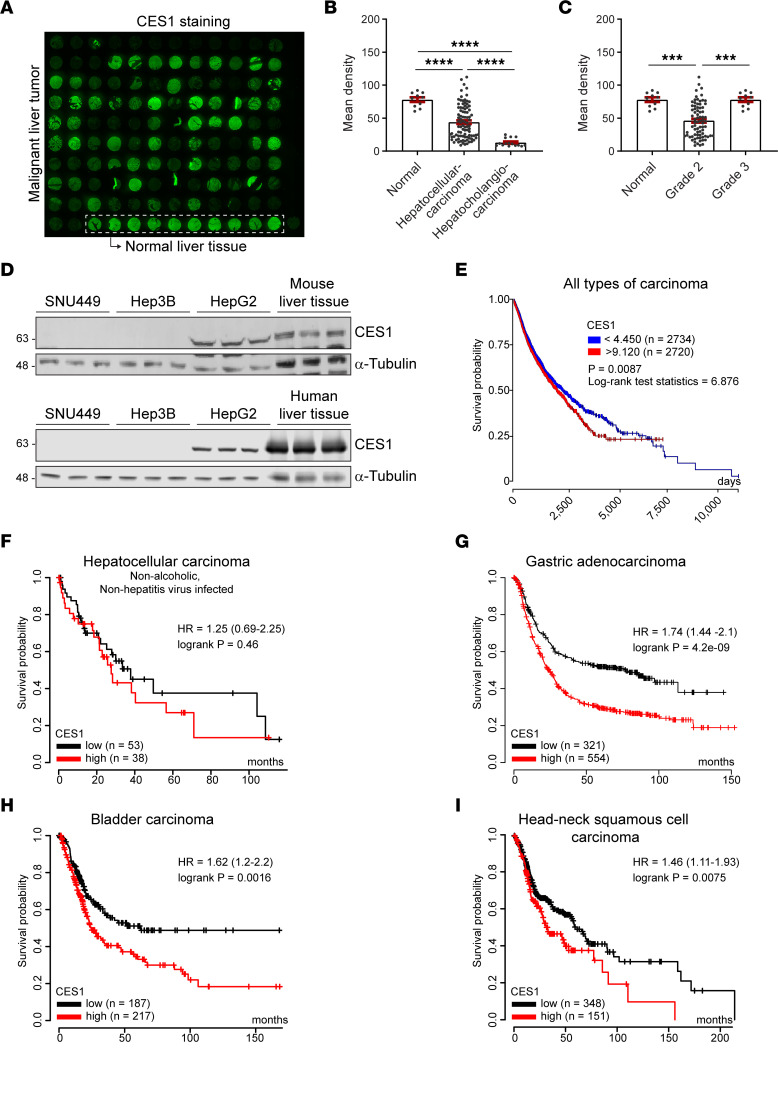
CES1 is selectively expressed in HCC and is associated with survival among liver cancer patients. (**A**) Immunofluorescence (IF) staining of CES1 in human liver tissue array. The array sections contained both normal liver tissues (10 samples at the bottom right in the dashed rectangle) and liver carcinoma samples. (**B** and **C**) Analysis of the fluorescence intensity of CES1 staining in **A** in different groups (***P < 0.001, ****P < 0.0001). (**D**) Western blot analysis of CES1 expression in HCC cell lines, mouse liver, and healthy human liver tissue. α-Tubulin was used as the loading control (*n* = 3 per group; representative of 3 repeats). (**E**) Correlation analysis between the expression levels of CES1 and survival rate among cancer patients from the TCGA PAN-Cancer (PANCAN) database. Data were generated using UCSC Xena with relatively large patient numbers (the numbers were shown in the panel). The cut-off values of the survival curve are the lower and upper quartiles of the CES1 expression; “low” or “high” are the lower and upper quartiles of the CES1 expression, respectively. (**F**–**I**) Correlation analysis between the expression levels of CES1 and survival rate among patients with different cancers. Data were generated using a KM plotter (kmplot.com) with relatively large patient numbers (the numbers were shown in each panel). The cut-off values of the survival curve are the lower and upper quartiles of the CES1 expression.

Previous studies have shown low to undetectable protein levels of CES1 in HCC and HCC-derived cell lines (38–40). However, with the newly developed high-affinity anti-CES1 antibodies, various levels of CES1 protein have been detected in different cancer cells, including HCC (www.proteinatlas.org/ENSG00000198848-CES1/pathology/liver+cancer#imid_19180094). Herein, we analyzed the levels of CES1 protein in an array of human liver cancer samples (*n* = 120) using immunofluorescence staining with the reported anti-CES1 antibody. The results revealed that the protein abundance of CES1 varied among different liver cancer patients (Figure 1A and Supplemental Figure 1A; supplemental material available online with this article; https://doi.org/10.1172/jci.insight.163624DS1). Overall, the CES1 protein was detectable in most HCC samples (*n* = 110). Among them, some were higher, while others were lower than those in normal livers (*n* = 10) (Figure 1A). Quantitative analysis further indicated that the average levels of CES1 protein in HCC were significantly lower than those in normal livers and that the levels in hepatocholangiocarcinoma were even lower than those in HCC (Figure 1B). Intriguingly, the levels in grade 2 HCC were decreased, while the protein increased to almost the same levels as in the normal liver in grade 3 HCC (Figure 1C). Further analysis of the different stages of HCC showed that the levels reduced when the tumors developed to an advanced stage (Supplemental Figure 1B). Interestingly, when analyzing the levels of CES1 in different HCC cell lines, we found that HepG2 cells synthesized CES1 at a level that was similar to normal mouse and human livers, whereas the protein in SNU449 and Hep3B was undetectable (Figure 1D).

We further analyzed CES1 expression in other cancer types based on the available databases (tnmplot.com, xena.ucsc.edu, and kmplot.com). First, we compared the expression levels of CES1 in normal and malignant human tissues (https://tnmplot.com/analysis/) (42). The results suggest that the liver expressed the highest levels of CES1 among all the tissues. Intriguingly, the mRNA levels were significantly upregulated in malignant liver tissue (Supplemental Figure 1C) — a result that is different from the results on the protein abundance (Figure 1, A and B). We then analyzed the correlation between the protein levels of CES1 and survival probability in different cancers. For the analysis, the cutoff values to define the levels with “low” or “high” are the lower and upper quartiles of the CES1 expression. The results indicate that the correlation between CES1 and survival probability in pancancers is weak (Figure 1E). However, there remained a trend toward a negative correlation between the levels of CES1 and the survival probability in nonalcoholic, nonhepatitis virus–infected HCC patients (Figure 1F). In several other cancer types, including gastric adenocarcinoma, bladder carcinoma, and head and neck squamous cell carcinoma if measured in enough cancer populations, the levels of CES1 were negatively correlated with the survival rate (Figure 1, G–I), while in other cancers, if measured in enough cancer populations, the levels of CES1 were positively correlated with survival rate (Supplemental Figure 1, D–U). In summary, our experimental and metaanalysis results suggest that CES1 is expressed at various levels in HCC and many other cancer types. They might play divergent roles in different types of cancers.

### Blockage of CES1 activity alters the dynamics of lipid droplets in HepG2 cells.

**Figure 2:**
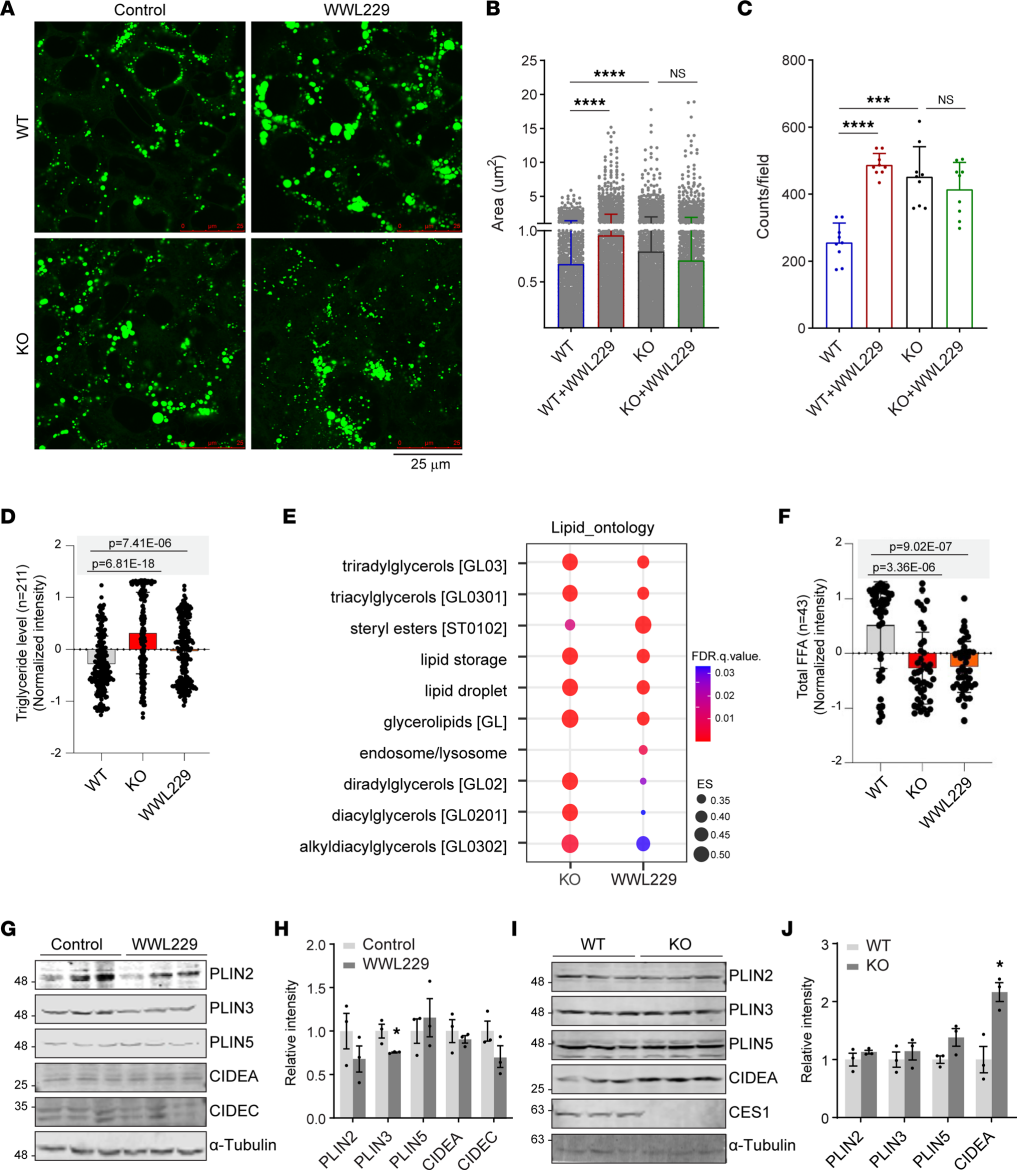
CES1 affects lipid droplets morphology and lipidomic profiles. (**A**) BODIPY staining of lipid droplets in the WT or *CES1*-KO (by a CRISPR deletion) HepG2 cells treated with or without 50 μM WWL229 for 48 hours. Scale bar: 25 μm.(**B**) Quantification of lipid droplet size in **A** (*n* = 5, data are represented as mean ± SD, Student’s *t* test, ****P < 0.0001). (**C**) Quantification of lipid droplet numbers in **A** (*n* = 5, data are represented as mean ± SD, Student’s *t* test, ***P < 0.001, ****P < 0.0001). (**D**) Lipidomic analysis for the total TG levels. Box plot shows the normalized intensity of TG (*n* = 211) in WT, KO, and WWL229-treated HepG2 cells (*n* = 3, data are represented as the mean ± SD, 1-way ANOVA followed by Dunnett T3-test) There is a correction for multiple comparisons using statistical hypothesis testing (*P* = 6.81 ***×*** 10^–18^, *P* = 7.41 ***×*** 10^–6^). (**E**) Dot plot showing the enrichment for lipid ontology from lipidomic analysis. (**F**) Box plot showing the normalized intensity of total FFA (*n* = 43) in WT, KO, and WWL229-treated HepG2 cells (*n* = 3, data are represented as the mean ± SEM, 1-way ANOVA followed by Dunnett T3-test) There is a correction for multiple comparisons using statistical hypothesis testing (*P* = 9.02 ***×*** 10^–7^, *P* = 3.36 ***×*** 10^–6^). (**G**) Western blot analysis of PLIN2, PLIN3, PLIN5, CIDEA, and CIDEC in HepG2 cells treated with or without 50 μM WWL229 for 48 hours. α-Tubulin was used as the loading control (*n =* 3 per group; representative of 3 repeats). (**H**) Quantification of the band intensity in **G** (*n* = 3 per group, each point represents a biological replicate). Data are presented as mean ± SD, Student’s *t* test (*P < 0.05). (**I**) Western blot analysis of PLIN2, PLIN3, PLIN5, CIDEA, and CES1 in WT and KO HepG2 cells. Particularly, the CES1 panel indicates the KO efficiency by a CRISPR deletion of CES1. α-Tubulin was used as the loading control (*n =* 3 per group; representative of 3 repeats). (**J**) Quantification of the band intensity in **I** (*n* = 3 per group; each point represents a biological replicate). Data are presented as mean ± SD, Student’s *t* test (*P < 0.05).

Given that we confirmed that liver cancer cells express varied levels of CES1, we sought to determine the role of CES1 in HepG2 cells, a well-characterized hepatoblastoma cell line that expresses high levels of CES1 (Figure 1D). Previously, we revealed that CES1 regulates the dynamics of lipid droplets by translocating onto their surfaces and digesting the lipid content in normal tissues (34, 35). Herein, BODIPY staining revealed that there were more lipid droplets with significantly larger sizes in the HepG2 cells when treated with WWL229, a specific inhibitor of CES1 (Figure 2, A–C). Similar results were observed in *CES1*-KO (by a CRISPR deletion) HepG2 cells (Figure 2, A–C). Interestingly, when treating *CES1*-KO cells with WWL229, the number and morphology of the lipid droplets did not further change, suggesting the specific inhibitory effect of WWL229 on CES1 activity. The results are in line with previous findings (34). To further characterize the changes in the lipid profiles upon CES1 inhibition, we performed untargeted lipidomic analysis using high-resolution mass spectrometry (MS) on cell lysates collected from WWL229-treated and *CES1*-KO HepG2 cells. The results show that TGs were globally increased, eventually leading to TG accumulation in WWL229-treated and *CES1*-KO cells (Figure 2D and Supplemental Figure 2A). Moreover, other components in the lipid droplets, such as stearyl esters, diradylglycerols, diacylglycerols, and alkyladiacylglycerols, were also increased or tended to increase (Figure 2E). In contrast, the levels of total FFA were significantly reduced in WWL229-treated and *CES1*-KO cells (Figure 2F). To determine whether loss of function of CES1 affects the dynamics of lipid droplets, we measured the levels of several key lipases and lipogenic enzymes that are related to lipid droplet dynamics. Western blotting results showed that lipid droplet–targeting lipolytic factors, such as ATGL, HSL, and CGI-58, did not significantly change (Supplemental Figure 2, B and C). Fatty acid synthetic and lipogenic factors, such as FASN and DGAT2, also did not change (Supplemental Figure 2, D and E). Intriguingly, while the total levels of the de novo lipogenic enzyme ACC1 were decreased, the ratio of phosphorylated ACC1/total ACC1 were slightly increased upon WWL229 treatment (Supplemental Figure 2, D and E). Similar results were observed in *CES1*-KO cells (Supplemental Figure 2, F–I), suggesting slowed progression of de novo lipogenesis upon loss of function of CES1 in the cells.

Next, we determined whether other lipid droplet–associated factors were altered. Western blotting results revealed that PLIN2 and PLIN3 were slightly decreased, while other factors, such as PLIN5, CIDEA, and CIDEC, were not significantly changed upon WWL229 treatment (Figure 2, G and H). In contrast, while all other factors remained unchanged, CIDEA levels were significantly increased in *CES1*-KO cells (Figure 2, I and J). In summary, altered morphology and dynamics of lipid droplets were observed in response to the blockage of CES1.

### Blockage of CES1 activity leads to impaired mitochondrial function.

**Figure 3:**
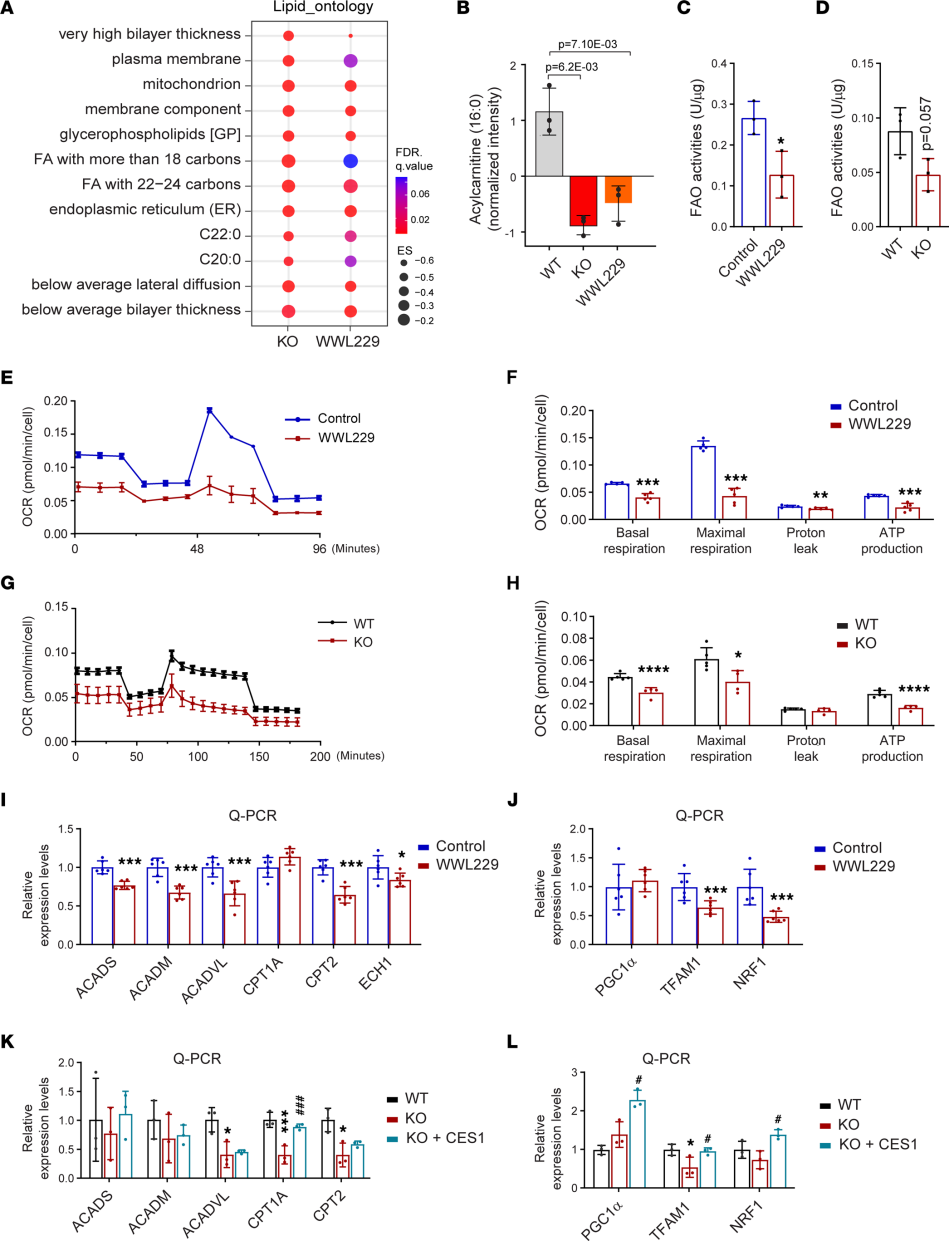
Blockage of CES1 impairs mitochondrial function. (**A**) Dot plot showing the enrichment for lipid ontology based on the lipidomic analysis. (**B**) Box plot showing the level of acylcarnitine (16:0) in WT, KO, and WWL229-treated HepG2 cells (*n* = 3). (**C**) Analysis of fatty acid oxidation in HepG2 cells treated with or without 50 μM WWL229 for 24 hours (*n* = 3).(**D**) Analysis of fatty acid oxidation in WT and KO HepG2 cells (*n* = 3). (**E**) Oxygen consumption rate (OCR) of HepG2 cells treated with or without 10 μM WWL229 analyzed by the Seahorse xFe24 instrument (*n* = 5). (**F**) Bioenergetic parameters inferred from OCR traces in **E** (*n* = 5). (**G**) OCR of WT and KO HepG2 cells analyzed using the Seahorse xFe24 instrument (*n* = 5). (**H**) Bioenergetic parameters inferred from OCR traces in **G** (n = 5). (**I**) qPCR analysis of β-oxidation–related genes in HepG2 cells treated with or without 50 μM WWL229 for 48 hours (*n* = 6). (**J**) qPCR analysis of mitochondrial biogenesis–related genes in HepG2 cells treated with or without 50 μM WWL229 for 48 hours (*n* = 6). (**K**) qPCR analysis of β-oxidation–related genes in WT, KO, and KO with reexpression of CES1 (KO + CES1) HepG2 cells (*n* = 3). (**L**) qPCR analysis of mitochondrial biogenesis–related genes in the WT, KO, and KO with reexpression of Ces1d (KO + CES1) HepG2 cells (n = 3). Each point represents a biological replicate. Data are represented as mean ± SD. Student’s *t* test for **B**, **C**, **D**, **F**, and **H**. One-way ANOVA followed by Dunnett T3 test for **K** and **J**. *P < 0.05, **P < 0.01, ***P < 0.001, ****P < 0.0001 versus WT or Control. ^#^*P* < 0.05, ^###^*P* < 0.001 versus KO.

CES1 hydrolyzes lipids to produce FFAs that serve as a fuel source for mitochondrial β-oxidation (34). The ontology of the lipidomic profiles indicated that blockage of CES1 activity by either WWL229 or *CES1* KO reduced the levels of lipid components involved in the plasma membrane, mitochondrial membrane, and endoplasmic reticulum (ER) membrane (Figure 3A). Interestingly, lipidomic analysis further revealed that the levels of acylcarnitine (16:0) were dramatically reduced, suggesting insufficient fatty acid β-oxidation (FAO) in WWL229-treated and *CES1*-KO cells (Figure 3B). The total levels of acylcarnitine also tended to be decreased in WWL229-treated and *CES1*-KO cells (Supplemental Figure 3A). In agreement with the lipidomic analysis, FAO activities were significantly reduced in WWL229-treated cells (Figure 3C), while they tended to be decreased in *CES1*-KO cells (*P* = 0.0573, Figure 3D). To determine whether loss of function of CES1 affects mitochondrial function, we monitored the oxygen consumption rate (OCR) by using a Seahorse XFe analyzer. The results revealed that key parameters, including the OCR of mitochondria, were massively decreased upon the addition of the modulators of respiration into WWL229-treated cells (Figure 3, E and F). Similar results were observed in *CES1*-KO cells (Figure 3, G and H). In line with the impaired mitochondrial function, quantitative PCR (qPCR) results revealed that the expression levels of mitochondrial oxidation enzymes, such as *ACADS*, *ACADM*, *ACADVL*, *CPT2*, and *ECH1* were significantly downregulated in the WWL229-treated HepG2 cells (Figure 3I). Consistently, other mitochondrial biogenetic genes such as *TFAM1* and *NRF1* were also downregulated (Figure 3J). Similar results were observed in *CES1*-KO HepG2 cells, while reexpression of CES1 recovered the gene expression levels to various degrees (Figure 3, K and L) in the KO cells. Notably, the protein levels of the mitochondrial respiratory complexes (I–V) were not altered in response to the inhibition of CES1 (Supplemental Figure 3, B and C). In conclusion, blocking CES1 activity impairs the respiratory function of mitochondria, which may affect the progression of tumor growth.

### Blockage of CES1 activity causes reduced levels of SCD in HepG2 cells.

**Figure 4:**
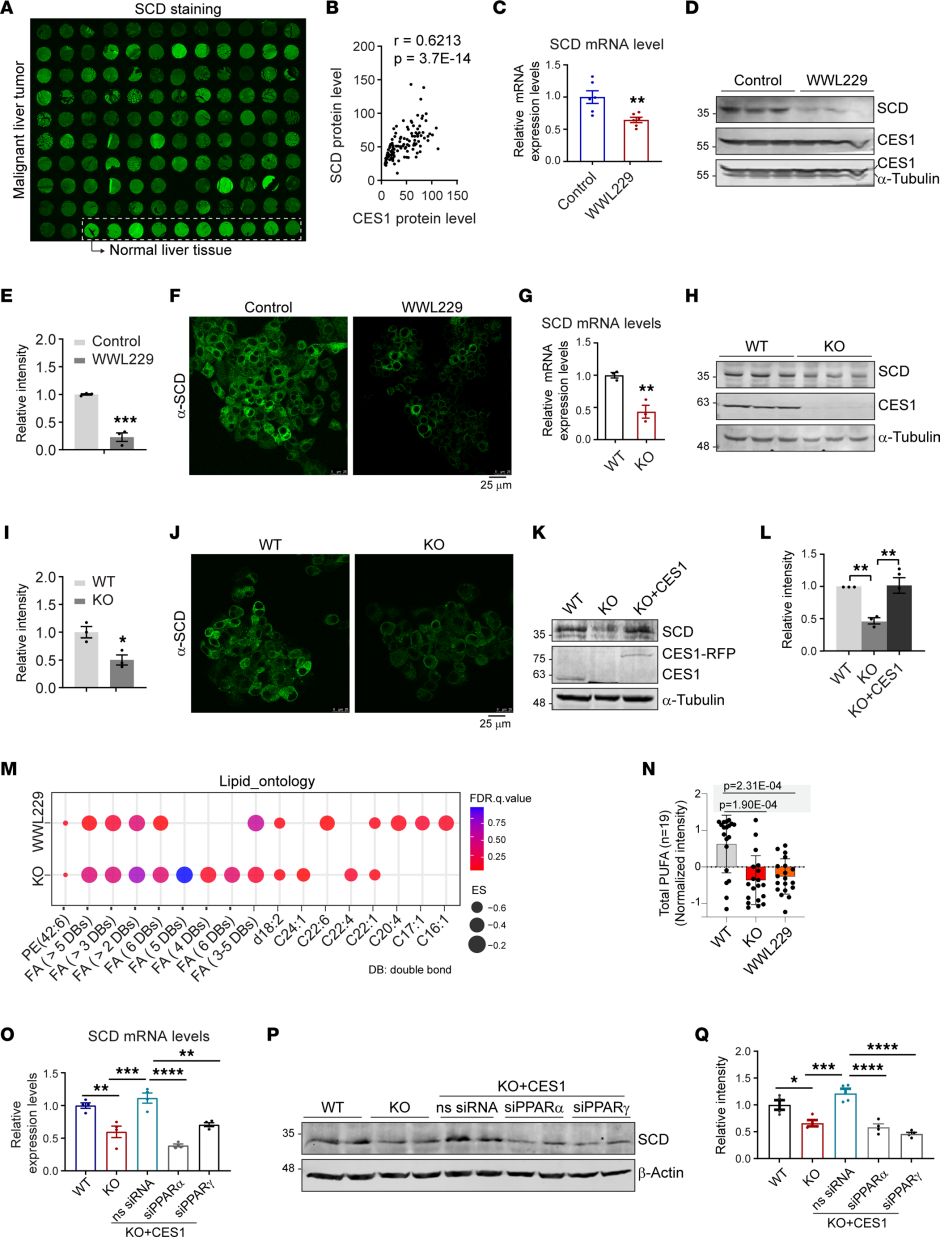
Blockage of CES1 activity decreases SCD levels through PPARα/γ. (**A**) IF staining of SCD in a human liver tissue array (the same tissue array as in Figure 1A). (**B**) Pearson’s correlation analysis of CES1 and SCD protein levels in the tissue array (**A** and Figure 1A). (**C**) qPCR analysis of SCD levels in HepG2 cells treated with or without 50 μM WWL229 for 48 h (*n* = 6). (**D** and **E**) Western blot analysis and quantification of SCD in the lysates from HepG2 cells treated with or without 50 μM WWL229 for 48 h (*n* = 3). (**F**) IF staining of the SCD on HepG2 cells treated with or without 50 μM WWL229 for 48 hours (representative of 6 fields, experiments were repeated 3 times). (**G**) qPCR analysis of SCD levels in WT and KO HepG2 cells (*n* = 3). (**H** and **I**) Western blot analysis and quantification of SCD and CES1 in lysates from WT and KO HepG2 cells. α-Tubulin was used as the loading control (*n* = 3). (**J**) IF staining of SCD on WT and KO HepG2 cells (representative of 6 fields; experiments were repeated 3 times). (**K** and **L**) Western blot analysis and quantification of SCD in lysates from WT, KO, and KO with reexpression of CES1 (KO + CES1) in HepG2 cells (*n* = 3). (**M**) Dot plot showing the enrichment for lipid ontology. (**N**) Box plot showing the level of total PUFA (*n* = 19) in WT, KO, and WWL229-treated HepG2 cells (*n* = 3). (**O**) qPCR analysis of SCD levels in different groups of HepG2 cells, including WT, KO, KO with reexpressed CES1 (KO + CES1), and reexpressed CES1 together with siPPARα (KO + CES1 + siPPARα) or siPPARγ (KO + CES1 + siPPARγ) (*n* = 3). (**P** and **Q**) Western blot analysis and quantification of SCD protein levels in different groups of HepG2 cells, including WT, KO, KO with reexpressed CES1 (KO + CES1), and reexpressed CES1 together with siPPARα (KO + CES1 + siPPARα) or siPPARγ (KO + CES1 + siPPARγ) (*n* = 4). Western blots are the representative of 3 repeats. Each point represents a biological replicate. Data are represented as mean ± SD. Student’s *t* test for **C**, **E**, **G**, and **I**. One-way ANOVA followed by Dunnett T3 test for **L**, **N**, **O**, and **Q**. *P < 0.05, **P < 0.01, ***P < 0.001, ****P < 0.0001.

SCD is a critical lipid-synthesizing enzyme. Its products, including MUFAs and multiple PUFAs, play essential roles in tumor cell proliferation and chemoresistance (28). Increased SCD has been demonstrated to correlate with tumor aggressiveness and poor patient diagnosis (9). Immunofluorescence analysis of an array of liver tumor samples with a specific anti-SCD antibody revealed that the protein levels of SCD varied among the different samples (Figure 4A). Importantly, we found a strong positive correlation between the protein levels of SCD (Figure 4A) and CES1 (Figure 1A and Figure 4B). Consistent with these results, qPCR analysis revealed that the expression levels of SCD were significantly downregulated upon WWL229 treatment in HepG2 cells (Figure 4C). Western blotting and immunofluorescence staining results further indicate that the protein levels of SCD were also reduced (Figure 4, D–F). Similarly, both the mRNA and protein levels were decreased in the *CES1*-KO cells, and reexpression of CES1 restored the levels of SCD in the KO cells (Figure 4, G–L). Interestingly, the rescuing effect of reexpressing CES1 was enhanced when cells were treated with FFAs such as oleic acid (OA) and palmitic acid (PA) (Supplemental Figure 4, A and B). The results of the lipidomic analysis are in line with the reduced levels of SCD upon blockage of CES1. Particularly, the lipid ontology of the global lipidomic profiles revealed that total PUFA levels were decreased in WWL229-treated and *CES1*-KO cells (Figure 4M). Notably, different species of PUFAs were reduced in WWL229-treated and *CES1-*KO cells at different levels (Figure 4M). Some MUFAs were also decreased in WWL229-treated and *CES1-*KO cells (Figure 4M). These results suggest that CES1 reprograms FFA saturation via regulation of SCD.

### PPARα/γ are involved in the CES1-mediated SCD regulation.

Next, we sought to define the mechanisms by which CES1 downregulates *SCD*. PPARα/γ has been reported to directly regulate SCD gene expression (43). Meanwhile, multiple PUFAs have been shown to activate the transcriptional activity of PPARα/γ (44). Interestingly, when analyzing the levels of FFAs by liquid chromatography–tandem MS (LC-MS/MS), we found that the total PUFAs were significantly diminished in WWL229-treated and *CES1*-KO cells (Figure 4N and Supplemental Figure 4C). We further found that blocking the functions of PPARα and/or PPARγ using their specific siRNAs abolished the recovery effects of CES1 on SCD expression in the *CES1*-KO cells, suggesting that PPAR α/γ mediates the function of CES1 on regulation of SCD (Figure 4, O–Q; refer to Supplemental Figure 4, D and E, for the PPARα/γ knockdown efficiency and Supplemental Figure 4F for the protein levels of CES1 reexpression). Intriguingly, the liver-enriched nuclear receptor HNF4α was also reported to be activated by lipid signaling triggered by CES1 (35). However, knockdown of HNF4α did not affect CES1-mediated SCD regulation in HepG2 cells (Supplemental Figure 4, G and H). Collectively, our results suggest that blockage of CES1 activity leads to downregulation of SCD. This effect is at least partially due to the diminished levels of PUFAs, which reduced PPARα/γ transcriptional activity on SCD expression.

### Blockage of CES1 activity induces ROS accumulation.

**Figure 5:**
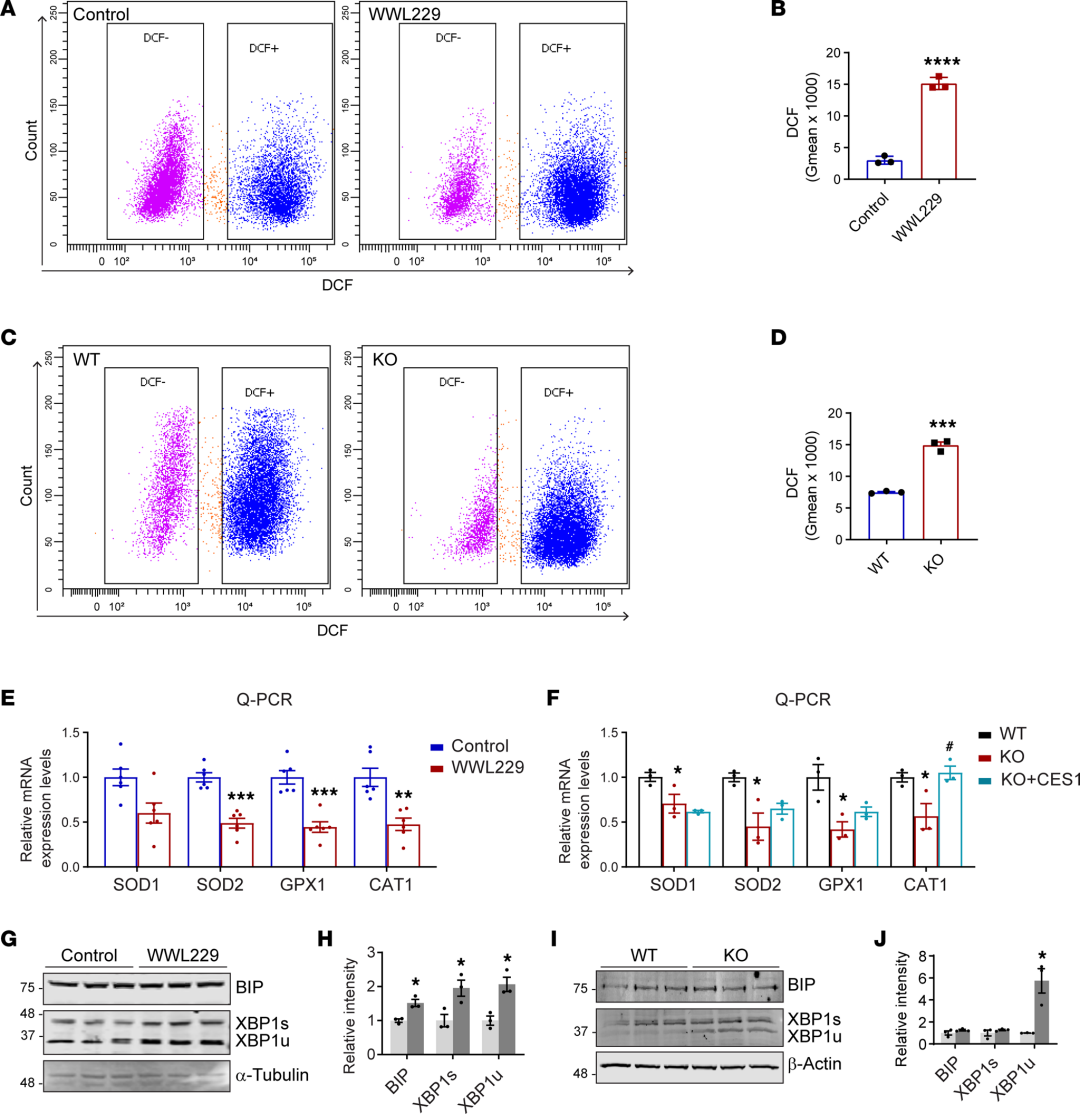
Blockage of CES1 activity induces ROS production and mild ER stress. (**A**) Measurement of cellular ROS production in HepG2 cells treated with or without 50 μM WWL229 for 48 h. ROS were detected with 2’, 7’-dichlorodihydrofluorescein (DCF) by flow cytometry. (**B**) Quantification of ROS production with geometric values (Gmean) in **A** (*n* = 3 per group, each point represents a biological replicate). Data are presented as mean ± SD, Student’s *t* test (****P < 0.0001). (**C**) Measurement of cellular ROS production in WT and KO HepG2 cells. ROS were detected with DCF by flow cytometry. (**D**) Quantification of ROS production with Gmean in **C** (*n* = 3 per group, each point represents a biological replicate). Data are presented as mean ± SD, Student’s *t* test (***P < 0.001). (**E**) qPCR analysis of antioxidant genes, including *SOD1*, *SOD2*, *GPX1*, and *CAT1*, in HepG2 cells treated with or without 50 μM WWL229 for 48 hours (*n* = 3 in each group; each point represents a biological replicate). Data are presented as mean ± SD, Student’s *t* test (**P < 0.01, ***P < 0.001). (**F**) qPCR analysis of antioxidant genes, including *SOD1*, *SOD2*, *GPX1*, and *CAT1*, in the WT, KO, and KO with reexpression of CES1 (KO + CES1) HepG2 cells (n *=* 3 in each group, each point represents a biological replicate). Data are represented as mean ± SD, 1-way ANOVA followed by Dunnett T3-test (*P < 0.05, versus WT, ^#^*P* < 0.05 versus KO). (**G**) Western blot analysis of ER stress–related proteins including BIP, XBP1s, and XBP1u in lysates from HepG2 cells treated with or without 50 μM WWL229 for 48 hours. α-Tubulin was used as the loading control (*n* = 3 per group; representative of 3 repeats). (**H**) Quantification of the band intensity in **G** (*n* = 3 per group; each point represents a biological replicate). Data are presented as mean ± SD, Student’s *t* test (*P < 0.05). (**I**) Western blot analysis of ER stress–related proteins, including BIP, XBP1s, and XBP1u, in lysates from WT and KO HepG2 cells. β-Actin was used as the loading control (*n* = 3 per group; representative of 3 repeats). (**J**) Quantification of the band intensity in **I** (*n* = 3 per group; each point represents a biological replicate). Data are presented as mean ± SD, Student’s *t* test (*P < 0.05).

Dysfunctional mitochondria and diminished SCD may lead to altered reactive oxygen species (ROS) levels (45, 46). Consistently, flow cytometry analysis revealed that WWL229-treated HepG2 cells exhibited a shift from 2’,7’-dichlorodihydrofluorescein (DCF, the ROS detector) to the DCF^+^ population when compared with the control cells (Figure 5A). Quantitative analysis showed a marked increase in the DCF^+^ cells after WWL229 treatment (Figure 5B). Similar results were observed in *CES1*-KO cells (Figure 5, C and D). In agreement with an increase in ROS, antioxidative enzymes, such as *SOD1*, *SOD2*, *GPX1*, and *CAT1*, were downregulated in WWL229-treated cells (Figure 5E). A similar effect was found in *CES1*-KO cells, and reexpression of CES1 back to the cells restored the expression of the enzymes (Figure 5F).

Both mitochondrial and SCD dysregulation leads to ER stress (47, 48). We next determined the pathological changes in the ER in response to blockage of CES1 activity in HepG2 cells. Western blotting results showed that the BIP and XBP1s/u proteins were increased in WWL229-treated HepG2 cells, suggesting ER stress in the cells (Figure 5, G and H). Intriguingly, while we observed the same alteration for the XBP1 proteins, the BIP levels were not altered in the *CES1*-KO HepG2 cells, suggesting some unidentified compensatory effect in the permanent KO cells (Figure 5, I and J). In conclusion, blockage of CES1 accelerated ROS production, while mildly inducing ER stress in HepG2 cells.

### Blockage of CES1 activity sensitizes the HCC to cisplatin treatment.

**Figure 6:**
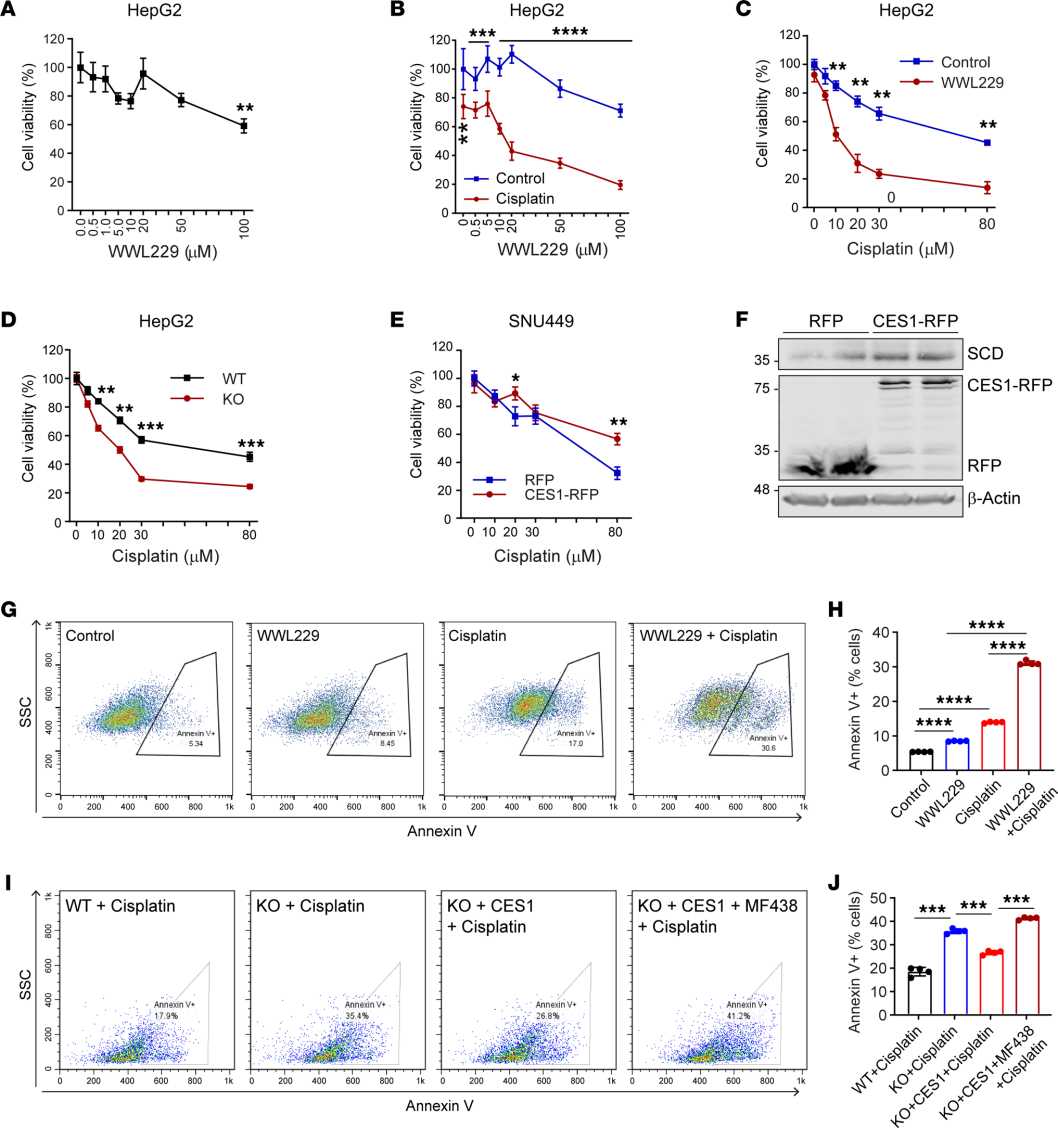
Blockage of CES1 activity sensitizes HCC for chemotherapy. (**A**) Cell viability analysis of HepG2 cells treated with different doses of WWL229 for 48 hours by MTT assay (*n* = 6). (**B**) Cell viability analysis of HepG2 cells cotreated with different doses of WWL229 and 20 μM cisplatin for 48 hours by MTT assay (*n* = 6). (**C**) Cell viability analysis of HepG2 cells cotreated with different doses of cisplatin and 50 μM WWL229 for 48 hours by MTT assay (*n* = 6). (**D**) Cell viability analysis of WT and KO HepG2 cells treated with different doses of cisplatin for 48 hours by MTT assay (*n* = 6). (**E**) Cell viability analysis of SNU449 cells treated with different doses of cisplatin for 48 hours by MTT assay (*n* = 6). (**F**) Western blot analysis of SCD, Red fluorescent protein (RFP, tag for transfected mouse CES1) in lysates from SNU449 cells transfected with CES1-RFP or RFP. β-Actin was used as the loading control (*n* = 2 per group; representative of 3 repeats). (**G**) Cell apoptosis assay of HepG2 cells treated with vehicle (control), 50 μM WWL229 (WWL229), 10 μM cisplatin (cisplatin), or a combination of 50 μM WWL229 and 10 μM cisplatin (WWL229 + cisplatin), for 48 hours. Apoptotic cells were detected using annexin V staining and quantified by flow cytometry. (**H**) Quantification of annexin V^+^ cells in **G** (*n* = 4 per group). (**I**) Cell apoptosis assay of WT HepG2 cells treated with 10 μM cisplatin (WT + cisplatin), KO HepG2 cells treated with 10 μM cisplatin (KO + cisplatin), KO with reexpression of CES1 with 10 μM cisplatin (KO + CES1 + cisplatin), or CES1 KO with reexpression of CES1 with 10 μM cis-platin and 10 μM MF438 (KO + CES1 + MF438 + cisplatin) for 48 hours, detected by annexin V staining with flow cytometry. (**J**) Quantification of annexin V^+^ cells in **I** (*n* = 4 per group). Three independent experiments with 6 technical replicates per experiment were performed in **A**–**E**. Each point represents a biological replicate. Data are represented as mean ± SD. Student’s *t* test for **A**–**E**. One-way ANOVA followed by Dunnett T3 test for **H** and **J**. *P < 0.05, **P < 0.01, ***P < 0.001, ****P < 0.0001.

A recent study reported that abnormal TG catabolism by CES1 promotes aggressive colorectal tumor growth (49). To determine whether loss of function of CES1 affects cell proliferation in HCC, we treated HepG2 cells with different doses of WWL229 and measured their viability using the MTT assay. Surprisingly, we only detected a mild effect on cell viability, even under the treatments of higher doses of WWL229 (20–50 μM) (Figure 6A). However, when cells were cotreated with the same doses of WWL229 and 20 μM cisplatin, a well-known anticancer agent, we found a synergistic effect of reduced cell survivability, even at relatively lower doses of WWL229 (0.5–5 μM) (Figure 6B). Moreover, when we chose a medium dose of WWL229 (50 μM) and cotreated the cells with different doses of cisplatin, we found that treatment with WWL229 markedly sensitized the cells to different doses of cisplatin (Figure 6C). A similar effect was observed in the *CES1*-KO cells (Figure 6D). Importantly, when we treated SNU449 cells (another liver cancer cell line that does not contain endogenous CES1) with cisplatin, we found a responsive cell killing effect. However, when we expressed a relatively low level of CES1 in them, the protein levels of SCD increased and the cells exhibited a resistance to cisplatin treatment (Figure 6, E and F). We further compared cell apoptosis in the groups by flow cytometry using annexin V^+^ cells. The results revealed that significantly more apoptotic cells were detected in the cotreated HepG2 cells than in the single treatment with either WWL229 (50 μM) or cisplatin (10 μM) (Figure 6, G and H). Consistently, more cleaved caspase 3, a marker of cell apoptosis, was detected in the cotreated cells (Supplemental Figure 5A). Similar results were observed in *CES1*-KO cells when treated with cisplatin (10 μM), while ablation of *CES1* itself did not induce dramatic cell apoptosis in the *CES1*-KO cells (Figure 6, I and J, and Supplemental Figure 5B). Notably, while reexpression of CES1 efficiently reduced the cell apoptosis induced by cisplatin in *CES1*-KO cells, blocking the activity of SCD by its specific inhibitor MF348 significantly abolished the rescuing effect of *CES1* reexpression (Figure 6, I and J), suggesting the key role of SCD in CES1-mediated HCC cell growth.

### Synergistic effect of CES1 inhibition and cisplatin treatment in HCC xenograft tumors.

**Figure 7:**
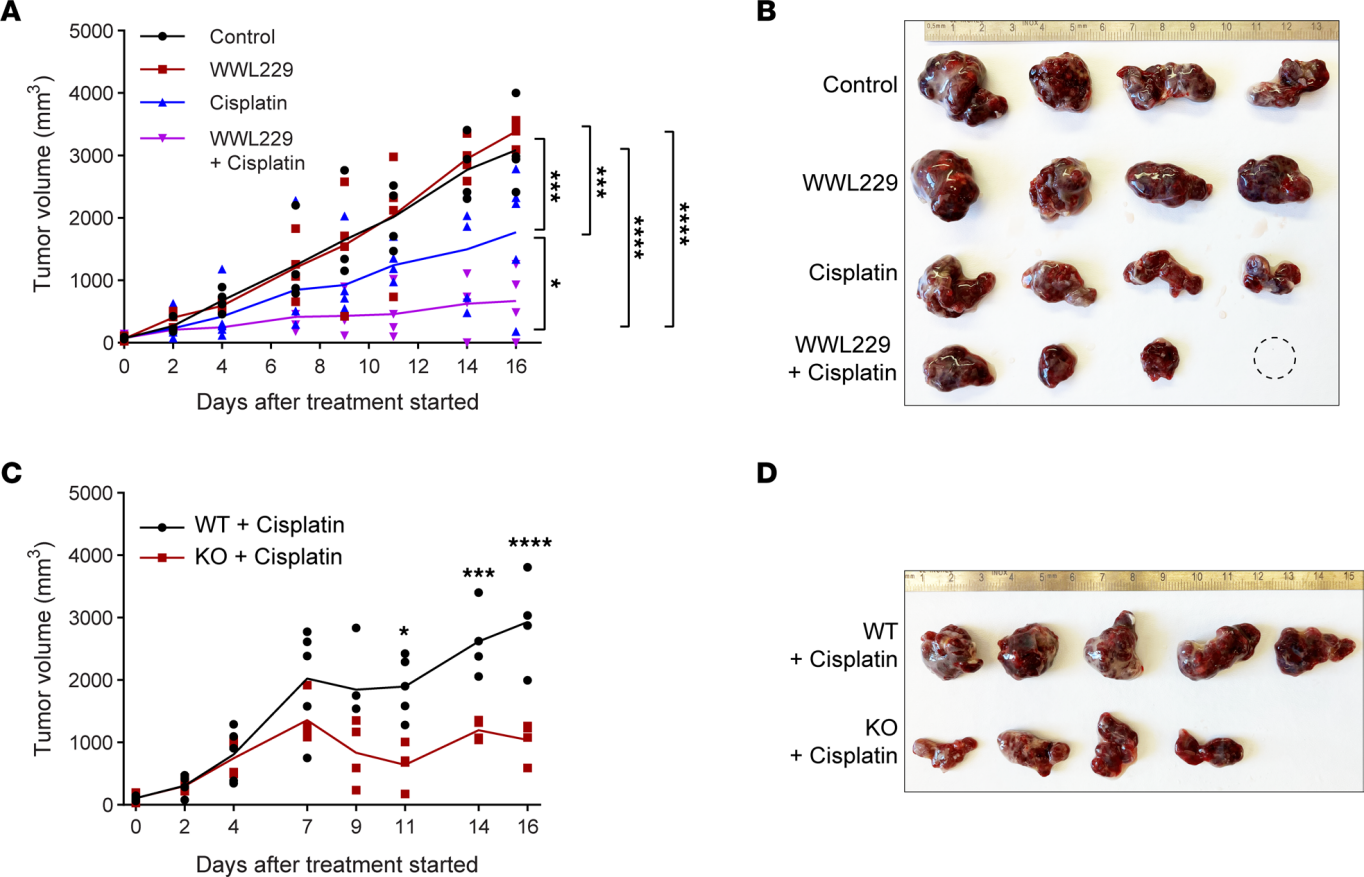
Blockage of CES1 activity significantly enhances cisplatin inhibition of tumor growth in HepG2 xenografted NU/J mice. (**A**) Measurement of tumor growth of xenografts formed by injected HepG2 cells in NU/J mice. The mice were treated with the vehicle, WWL229, cisplatin, or a combination of WWL229 and cisplatin for 16 days by i.p. injection (*n* = 4 per group; each point represents a biological replicate). Two-way ANOVA followed by Tukey multiple-comparison test (*P < 0.05, ***P < 0.001, ****P < 0.0001). (**B**) Images of xenograft biopsies collected from mice after treatment in **A**. The blank circle represents a completely shrunk xenograft tumor. (**C**) Tumor volumes of WT and KO HepG2 xenografts in NU/J mice treated with 10 μmol cisplatin/kg body weight for 16 days. Data shown are tumor growth curves (*n* = 5 or 4 per group; each point represents a biological replicate). Student’s *t* test (*P < 0.05, ***P < 0.001, ****P < 0.0001). (**D**) Images of xenograft biopsies collected from mice after treatment in **C**.

To further test the synergistic effect of loss of function of CES1 and treatment of cisplatin, we performed a tumor growth assay in HepG2 xenografted NU/J mice. The results indicate that, while single treatment of WWL229 (155 μmol/kg) did not have a significant effect and while and single treatment of cisplatin (10 μmol/kg) only slightly inhibited the growth of xenograft tumors, cotreatment with WWL229 and cisplatin significantly inhibited tumor growth (Figure 7A). In agreement with the dynamic changes of the tumor growth during the cotreatment, morphological examination of the xenograft tumors after the treatments showed that the sizes of the xenografts collected from the cotreated mice were dramatically smaller than those of the single-treated groups (Figure 7B; 1 sample in the cotreated samples shrank to an undetectable size, as shown by the dashed circle). A similar inhibitory effect on xenograft growth was observed in *CES1*-KO HepG2 cells when treated with cisplatin (10 μmol/kg), while ablation of *CES1* itself did not affect the *CES1*-KO xenograft tumor growth (Figure 7, C and D, and Supplemental Figure 6, A and B). Consistently, we detected a decreased ratio of phosphorylated AKT and total AKT in *CES1*-KO xenografts, suggesting reduced tumor growth (Supplemental Figure 6, C and D). Notably, we did not find any differences in body weights between the groups during the entire treatment process (data not shown).

Sorafenib is another chemotherapeutic agent used to treat advanced HCC (50, 51). Interestingly, a correlation analysis using the KM plotter (kmplot.com) demonstrated a trend toward a negative association between the levels of CES1 and survival probability in HCC patients treated with sorafenib (Supplemental Figure 6E), suggesting a potential synergistic effect between CES1 inhibition and sorafenib treatment. However, more experimental and preclinical studies are needed to test this hypothesis.

### A working model for the role of CES1 in HCC growth.

**Figure 8:**
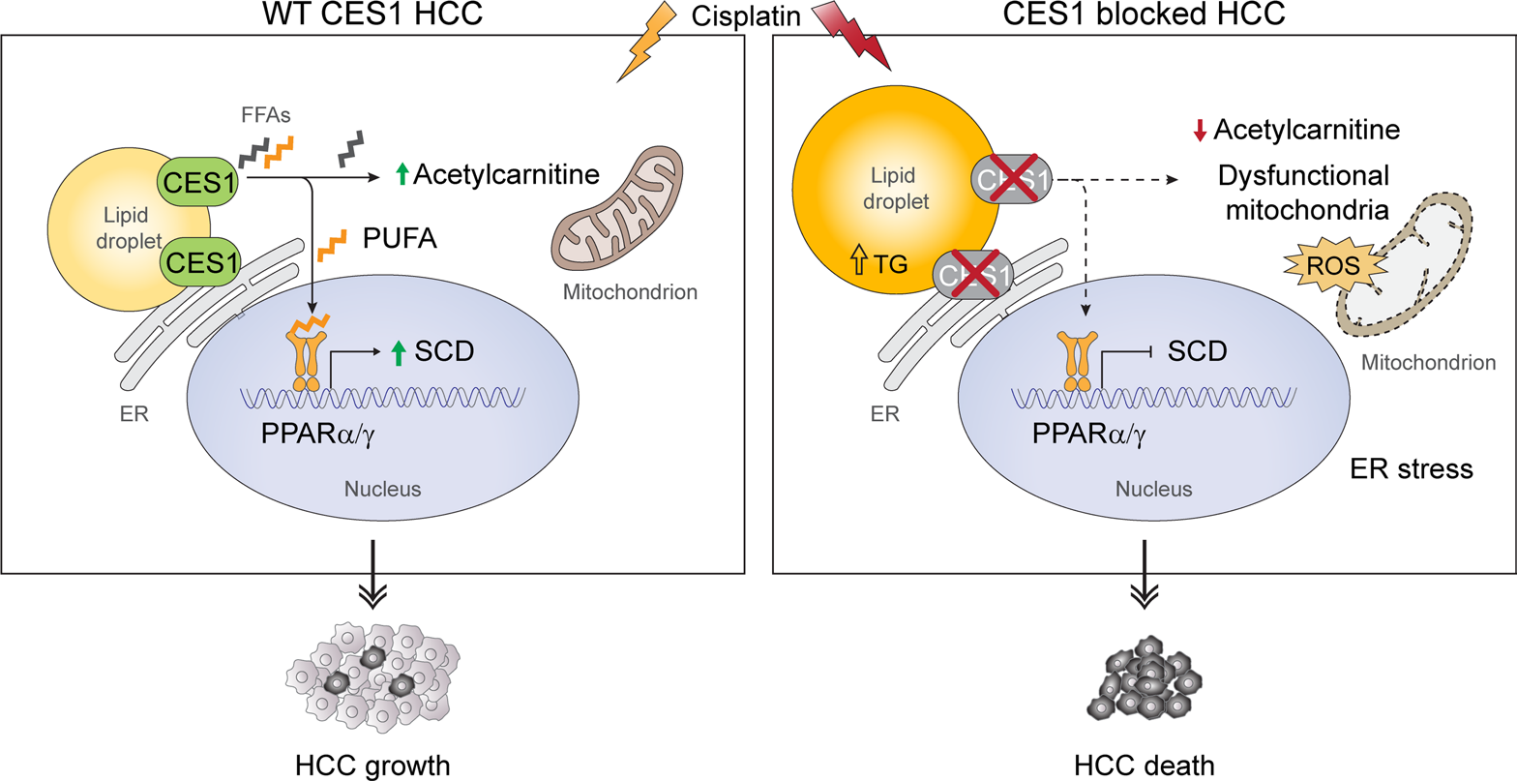
Working model. A proposed working model of CES1 regulation and function in lipid metabolism and tumor growth.

A working model is proposed based on our findings in this study. In the model, FFAs produced by CES1 fuel the mitochondria for β-oxidation and ATP production to support tumor growth. Meanwhile, some unique FFAs produced by CES1, such as multiple PUFAs, may function as signaling molecules for PPARα/γ activation. Upon activation, PPARα/γ binds to the SCD promoter and, hence, upregulates its expression. Upregulated SCD further promotes tumor growth by decreasing ER stress and increasing the levels of phosphorylated AKT and other pathways. Consequently, the enhanced mitochondrial function and increased levels of SCD induced by CES1 activation promote tumor growth and potential chemoresistance (Figure 8, left). In contrast, blockage of CES1 activity by pharmacological or genetic approaches impairs mitochondrial function, increases ROS production, and decreases the levels of SCD, thereby sensitizing HCC to chemotherapeutic agents, such as cisplatin and sorafenib (Figure 8, right).

## Discussion

Lipid metabolism reprogramming has drawn considerable attention as an essential factor in tumor development and progression (17, 52). CES1 is a key enzyme that plays an important role in lipid metabolism (33). In humans, CES1 is abundantly expressed in the normal liver and adipose tissues, where it plays an important role in lipid droplet metabolism, lipoprotein assembly, and secretion (33, 36, 53). However, for a long time, it was considered to be undetectable in HCC and human liver cancer cell lines (38, 39, 54, 55). Although artificially transfected CES1 exerted antiproliferative effects in liver cancer cell lines (38), the bona fide role of CES1 in HCC per se has not yet been well characterized. In this study, we have demonstrated that CES1 was selectively expressed at various levels in different liver cancer samples and HCC cell lines. Intriguingly, while the mRNA levels of CES1 are upregulated consistently in all the liver tumors, its protein levels exhibit high heterogeneity among different types of HCC, suggesting profound posttranscriptional regulations of CES1. Of note, the trend toward a negative correlation between the levels of CES1 and the progression of HCC suggests a potential role of CES1 in NAFLD-induced HCC development. Further studies are needed to confirm the hypothesis. More importantly, for the first time to our knowledge, we report that, while blockage of CES1 only had a mild effect on inhibition of HCC growth and expansion, it potently sensitized HCC cells to chemotherapeutic agents, such as cisplatin. Mechanistically, we discovered that blockage of CES1 caused TG accumulation and, hence, induced the rewiring of lipid metabolism, which eventually led to impaired mitochondrial function. Moreover, we identified the CES1-PPARα/γ-SCD axis as a key modulator. Specifically, lack of unique FFAs — especially multiple species of PUFAs, due to blockage of CES1 — suppressed the transcriptional activity of PPARα/γ on SCD expression, which ultimately increased the sensitivity of HCC to the treatment of cisplatin.

Emerging evidence has demonstrated that dysregulation of dynamics of lipid droplets in nonadipocytes is closely related to tumor cell adaptability and progression (56). In particular, a slower turnover rate of lipid droplets by targeting lipolysis has been demonstrated to be detrimental to tumorous cells, providing a potential therapeutic opportunity to treat cancer (56, 57). In that context, we and others have recently revealed that CES1 targets lipid droplets and hydrolyzes their surface lipid contents (34, 37, 58). In this study, we found that blockage of CES1 by WWL229 or genetic KO induced accumulation of more lipid droplets with larger sizes in HCC. Although the levels of other lipid droplet–surrounding factors, including ATGL, HSL/phosphorylated HSL, GCI-58, and CIDEA–C, did not significantly change, the de novo lipogenic enzyme ACC decreased slightly, while the ratio of phosphorylated ACC/total ACC increased upon blockage of CES1, suggesting that lipogenesis was also affected by inhibition of CES1 in HCC.

Functional mitochondria are important for cell growth. Specifically, mitochondria control diverse vital parameters, such as the generation of energy through oxidative phosphorylation, regulation of ROS and oxidative stress, and initiation of apoptosis in aggressively growing cancerous cells (59). Enhanced mitochondrial metabolism alters cell redox status and increases ROS generation, which further stimulates tumor cell proliferation. Particularly, mitochondrial FAO plays multifaceted roles in proliferation, survival, stemness, and chemoresistance of the cancerous cells (60). Accelerated lipolysis on lipid droplets significantly increases the levels of the FAO and enhances the function of mitochondria to promote cancer cell growth, which has been highlighted as a “lipolytic phenotype” (16, 60). Herein, we found that blockage of CES1 activity leads to formation of larger lipid droplets and slowed lipolysis, as evidenced by lower levels of FFAs and glycerol in WWL229-treated and *CES1*-KO HepG2 cells. Dysregulation of lipolysis further reduced the production of total FFAs and a transport form of fatty acids acylcarnitine, thereby impairing the mitochondrial energy production function, as demonstrated by the significantly lower OCRs during the Seahorse assay. Moreover, the expression levels of FAO-related enzymes were also downregulated, probably due to the negative feedback from the lack of FFAs in the mitochondria. The metabolic rewiring in the mitochondria bears a potential to sensitize cells to chemotherapeutic drugs. Given the fact that CES1 inhibition also impairs the mitochondrial function in normal cells (34, 61), caution should be taken when considering the clinical implication of CES1 inhibitors in future.

Tremendous studies have demonstrated that the lipogenic factor SCD is involved in cancer cell proliferation and metastasis (9). SCD levels are positively correlated with cancer aggressiveness and chemoresistance (9). Mechanistically, its direct products, such as numerous MUFAs and PUFAs, exert their impact on tumorigenesis via enhanced phosphorylation of AKT and decreased ER stress (9, 62). Targeting SCD activity by its specific inhibitors results in tumor-specific apoptosis (63). In addition, identification of novel signaling pathways that downregulate its expression might provide alternative strategies to treat or prevent SCD-associated malignant disease. In this context, we reported that blockage of CES1, either pharmacologically or genetically, reduced SCD levels in HCC. More importantly, our analysis on clinical data revealed a strong correlation between the levels of CES1 and SCD in patients with liver cancer. Lipidomic profiles indicated that blockage of CES1 led to decreased levels of numerous MUFAs and PUFA (*n* > 2 double bonds), which was consistent with the reduced levels of SCD. Intriguingly, not all species of MUFA were decreased in response to the inhibition of CES1, reflecting the complexity of the regulation of lipid synthesis and metabolism at multiple levels. We further detected increased ROS generation and ER stress — results that are also in agreement with the reduced levels of SCD in CES1-blocked HepG2 cells. Importantly, treatment with the SCD-specific inhibitor MF438 efficiently blocked the CES1 effect on HCC apoptosis, further highlighting the key role of SCD in CES1-mediated cancer cell growth.

We further investigated the mechanisms underlying the regulation of SCD by CES1. Previous studies reported that SCD is a direct target of PPARα/γ (43, 44, 64, 65). In this study, targeting PPARα/γ molecules by their specific siRNAs demonstrated their key role in CES1-mediated SCD regulation. To address how CES1 manipulates the transcriptional activation of PPARα/γ, we performed LC-MS/MS analysis on WWL229-treated and *CES1*-KO HepG2 cells and identified multiple PUFAs that were diminished in the cells. These PUFAs have been demonstrated to be the endogenous ligands for PPARα/γ activation (44). Interestingly, another liver-specific nuclear receptor, HNF4, has also been reported to regulate SCD expression (66). Indeed, we recently revealed that HNF4 is involved in the development of liver steatosis caused by loss of function of Ces1 in diet-induced obese mice (35). However, in this study, we did not find evidence that supported the involvement of HNF4 in CES1-mediated regulation of SCD in HCC. The difference of HNF4 functions in normal hepatocytes and HCC demonstrate its cell type–specific regulation of lipid signaling. Notably, PUFAs have been demonstrated to regulate SCD at both the expression and protein levels (67), suggesting that, in addition to the regulation of expression through PPAR α/γ, decreased PUFAs might also affect the levels/activity of SCD via other profound mechanisms in HCC. Of note, in addition to the identified PPARα/γ regulation, other lipogenesis pathways, such as SREBP1/2 signaling, might be also involved in the regulation of SCD mediated by CES1. Further studies are needed to test these pathways.

Unexpectedly, even though blocking CES1 activity impairs mitochondrial function and reduces SCD levels, both of which might vitally affect the growth and proliferation of tumor cells, we only observed a mild effect of cell apoptosis in WWL229-treated and *CES1*-KO HepG2 cells. However, when we combined the approaches of CES1 blockage and administration of the anticancer agent cisplatin, we detected a synergistic effect of cell apoptosis and tumor inhibition in HCC. Furthermore, the combination treatment significantly reduced HCC xenograft tumors in NU/J mice. Our findings are of clinical significance. As we know, emerging evidence has demonstrated the chemotherapeutic potential of cisplatin in the treatment of patients with advanced HCC. Unfortunately, despite a certain level of therapeutic efficacy, significant numbers of HCC patients have experienced insensitivity or resistance to cisplatin administration, eventually leading to therapeutic failure (7). Rewiring of lipid metabolism has been recognized as a major cause of chemoresistance in HCC to cisplatin. Indeed, a better understanding of the key role of lipid metabolism in HCC has changed the concept about the cancer from a “genetic disease” to a “metabolic disease” (68). To support this notion, several lipid metabolic enzymes, including SCD, ACSS2, ACC1/2, and alkylglyceronephosphate synthase (AGPS), have been reported to be directly involved in cisplatin resistance (69, 70). Moreover, inhibition of the lipid synthesis enzyme fatty acid synthetase (FASN) efficiently reversed cisplatin resistance in cancer cells (71). In our study, we found that blockage of CES1 led to alterations in cisplatin resistance–related factors, including PUFAs, SCD, and ACC1, thereby interfering with lipid metabolism and sensitizing HCC to cisplatin treatment. Our findings, thus, provide a strategy to deal with the chemoresistance of HCC in clinic. Further mechanistic studies are warranted to elucidate the association between lipid metabolism and DNA damage and repair induced by cisplatin in HCC. Importantly, our analysis of the clinical database further suggested that CES1 levels tended to have a negative association with the survival rate of sorafenib-treated HCC, suggesting that targeting CES1 might have the potential to sensitize HCC to a broad range of chemotherapeutic agents.

In conclusion, HCC has been demonstrated to be naturally or adaptively resistant to chemotherapeutic agents, including cisplatin, thereby leading to uncontrolled tumor growth and metastasis during chemotherapy. Our findings demonstrate that targeting the CES1-PPARα/γ-SCD axis may sensitize HCC tumors to cisplatin and other anti-HCC drugs. Therefore, interfering with lipid metabolism by blocking CES1 activity has great potential for the treatment of HCC.

## Methods

Supplemental Methods are available online with this article.

### Analysis of CES1 expression in human samples.

CES1 expression analysis in normal human and tumor tissues was performed using TNMplot (https://tnmplot.com/analysis/). The significant difference between the normal and tumor tissues was analyzed using the Mann-Whitney *U* test, which was conducted using the web tool. Correlation analysis of CES1 expression and overall survival in all types of cancer was conducted using the tool in UCSC Xena browser (http://xena.ucsc.edu/) with the TCGA Pan-Cancer databases. Correlation analyses of CES1 expression and survival in different cancer types were performed using KM plotter (https://kmplot.com/analysis/). For the analysis, the cutoff values to define the levels with “low” or “high” are the lower and upper quartiles of the CES1 expression, respectively.

### Tissue array and protein level analysis.

Liver carcinoma and normal tissue arrays were obtained from the US Biomax (no. BC03119b). The protein levels were analyzed by immunofluorescence staining with an anti-CES1 antibody. Briefly, deparaffinized slides containing the tissue arrays were permeabilized with 0.2% Triton X-100 in 1× PBS for 10 minutes and incubated with sodium citrate buffer at 95°C for 30 minutes for antigen retrieval. After blocking with 5% BSA for 1 hour, the slides were incubated with primary antibodies at 4°C overnight. The slides were then washed with 1× PBST 3 times (0.1% Tween-20 in PBS) and further incubated with Alexa Fluor 488–conjugated donkey anti–rabbit IgG (catalog 711-545-152, Jackson ImmunoResearch) at room temperature for 1 hour. After incubation, the slides were washed with 1× PBST for 3 times and mounted. The slides were imaged using a Cytation 5 imaging reader. Anti-CES1 antibody (catalog HPA012023, Sigma-Aldrich) and anti-SCD antibody (catalog HPA012107, Sigma-Aldrich) were used for immunofluorescence staining.

### Animals and in vivo xenograft model.

NU/J mice (no. 002019) were purchased from The Jackson Laboratory and housed in an animal facility with a 12-hour light/dark cycle at room temperature (22°C ± 1°C). The animals had free access to water and regular chow diet. When mice were 10 weeks old, 5 × 10^6^ HepG2 cells (WT and *CES1* KO) were s.c. injected into the right flanks of the mice. Tumor volumes were measured with a caliper 3 times per week and calculated using the formulation of 0.5 × length × width^2^. When the volumes reached approximately 100 mm^3^, WWL229 (155 μmol/kg body weight) and cisplatin (10 μmol/kg body weight) were i.p. administered to the mice 3 times a week for a total of 2 weeks. Appropriate vehicles (1% dimethyl sulfoxide, 24% polyethylene glycol 400, and 6% Tween-80 in PBS for WWL229 cells and PBS for cisplatin cells) were administered to the control mice. After 16 days of drug treatment, the animals were sacrificed and the tumor tissues were collected for further analysis.

### Statistics.

All data are presented as the mean ± SEM or mean ± SD. All statistical analyses were performed using GraphPad Prism 8. The unpaired 2-tailed Student’s *t* test or 2-tailed Mann-Whitney *U* test was used to compare the differences between the 2 groups in the meta-analysis. One-way ANOVA was used to compare the differences among multiple experimental groups. The Dunnett *T3* test was applied for the post hoc test. There is a correction for multiple comparisons using statistical hypothesis testing. Two-way ANOVA followed by Tukey multiple-comparison test was used to for the tumor volume comparison. Pearson’s correlation was used to analyze the relationship between CES1 and SCD protein levels in the tissue array samples. Statistical significance was set at *P* < 0.05. For the lipidomic analysis, raw peak intensity was represented by normalized *Z* scores, and pairwise *P* values were calculated using 2-tailed Student’s *t* test and 2-tailed unequal variations to convey the significant abundance in the treated groups compared with the controls.

### Study approvals.

The protocol for the animal experiments was reviewed and approved by the Animal Welfare Committee of the University of Texas Health Science Center at Houston (Animal protocol no. AWC-21-0019).

## Author contributions

KS, PLL, and RL conceptualized the research; KS, GL, XL, PLL, KLEM, and RL designed research studies; GL, XL, JY, BF, SW, and BW conducted experiments; GL, XL, IM, and BW acquired data; KS, GL, XL, IM, KLEM, and PLL analyzed data; KS, GL, and XL wrote the manuscript; and GL, XL, IM, JY, BF, BW, KLEM, PLL, and RL edited the manuscript.

## Supplementary Material

Supplemental data

Supplemental table 1

## Version 1 12/06/2022

In-Press Preview

## Version 2 01/24/2023

Electronic publication
