# Supplementary material for: Interfering with lipid metabolism through targeting CES1 sensitizes hepatocellular carcinoma for chemotherapy
Source: JCI Insight. 2023 Jan 24;8(2):e163624. doi: 10.1172/jci.insight.163624 (PMC9977307; doi:10.1172/jci.insight.163624)
Supplement: Supplemental data [file jciinsight-8-163624-s135.pdf]

Supplemental Materials and Methods

Cell culture and drug treatments

HepG2, Hep3B, and SNU449 cells were obtained from American Tissue Culture Collection (ATCC, Manassas, VA). All the cell lines were cultured in complete DMEM media (ATCC, #30-2003) supplemented with 10% fetal bovine serum, 1 x antibiotic-antimycotic and 10 mM HEPES. The cells were cultured in a humidified atmosphere of 5% of CO_2 at 37°C. To block CES1 function, cells were treated with 50 µM WWL229 for 48 hours unless otherwise indicated. For the seahorse experiment, cells were pre-treated with 10 µM WWL229 for 15 min before the analysis and continuously treated with WWL229 during the entire analysis. For the Annexin V analysis, HepG2 cells were treated with 20 µM Cisplatin or 5 µM MF438 for 48 hours before harvest for analysis. Where indicated, cells were treated with 0.2 mM BSA conjugated oleic acid or 0.1 mM BSA conjugated palmitic acid for 12 hours. Each biological replicate was multiple-well analysis at the same time. Meanwhile, the related experiments were repeated for three times.

Generation of CES1 KO cell lines

To generate CES1 KO HepG2 cell lines, gRNA1(5'-GGCCACTCTCTCTGCTTCCG-3') and gRNA2(5'-TGCCTTTATCCTGGCCACTC-3') targeting CES1 were cloned into the BbsI site of the CRISPR/Cas9 vector (pSpCas9n(BB)-2A-Puro, Addgene #62988). Upon verification of the sequence, the plasmids were co-transfected into HepG2 cells. Thirty-six hours after transfection, the cells were treated with 7 ng/ml puromycin to select the transfected cells. Three days later, the cells were trypsinized and seeded into 100 mm^2 plates (100 cells/plate) to achieve single-cell colonies. The CES1 KO efficiency of each colony was determined by western blotting with an anti-CES1 antibody. In the end, two colonies with complete CES1 KO were selected for the study.

Transfection of plasmids by electroporation

The plasmid of RFP-CES1d was constructed as follow: the cDNA of mouse CES1d was amplified from eGFP.CES1d by PCR using Pfu DNA polymerase and ligated into the pcDNA3-mRFP (Addgene #13032) between EcoRI and NotI restriction sites. The cloned construct was verified by Sanger sequencing (Integrated DNA Technologies).

Cells were transfected by electroporation with GenePulser Xcell system (Bio-Rad) following the manufacturer's protocol. Briefly, 2 x 10^6 suspended HepG2 cells in OPTI-MEM were electroporated at 160V and 600 µF capacitance with 20 µg indicated plasmids or 10 µmol siRNA in a 0.2 mm cuvette. The transfected cells were then reseeded into well plate and cultured for 48 hours before harvest for further

analysis. siPPARα (sc-36307), siPPARγ (sc-29455), or scrambled siRNA were used in this study. Each biological replicate was multiple-well analysis at the same time. Meanwhile, the related experiments were repeated for three times.

Cell viability assay

The cell viability was determined by 3-[4,5-dimethylthiazol-2-yl]-2,5-diphenyltetrazolium bromide (MTT) assay. Cells were seeded in 96-well plates at the density of 5,000-10,000 cells per well for 24 h followed by indicated treatments for another 48 h. After the incubation period, MTT labeling reagent was added into the wells at a final concentration of 0.5 mg/ml and incubated for 4 h. MTT solvent (4 mM HCl, 0.1% NP40 in isopropanol) was then added into each well and incubated for 15 min at room temperature. The absorbance was measured at 590 nm. Each biological replicate was multiple-well analysis at the same time. Meanwhile, the related experiments were repeated for three times.

ROS analysis

The production of ROS was estimated with the fluorescent dye 2',7'-dichlorodihydrofluorescein diacetate (H_2DCFDA) (Thermo Fisher Scientific, #D399). After treatments as indicated, the cells were detached and incubated in 10 mM cell-permeant H_2DCFDA for 60 min at 37°C. Afterwards, the cells were washed 3 times with PBS to remove the free fluorescent dye and then subjected to flow cytometry analysis. Data were acquired using a BD FACS ARIA II SORP flow cytometry (BD, Biosciences) and analyzed using FlowJo software.

Apoptosis analysis

Cell apoptosis was tested by Annexin V staining. After treatments as indicated, the cells were stained with Annexin V-FITC (BD Pharmingen, #560931) for 15 min and washed with PBS to remove free dye. The stained cells were counted by flow cytometry analysis.

Mitochondrial function assays

Oxygen consumption rates (OCR) of the mitochondria in HepG2 cells were measured by a Seahorse XFe24 Analyzer (Agilent Technologies). Briefly, cells were first seeded and cultured in XFe24 cell culture plates. Upon experiment, the medium was replaced with pre-warmed assay medium (Seahorse base medium supplemented with 1 mM pyruvate, 10 mM glucose, and 2 mM glutamine, pH = 7.4) and the plate were incubated at 37°C in a non-CO_2 incubator for 1 h. Next, the plate was transferred to the Seahorse XFe24 to record the OCR of the cells at different time windows. The following compounds were subsequently injected into assay medium to assess cell mitochondrial function: 5 μM oligomycin, 1 μM carbonyl cyanide

4-(trifluoromethoxy) phenylhydrazone (FCCP), and a mixture of 5 µM rotenone and 15 µM antimycin A. 10 µM WWL229 was added through port A 15 min prior to the oligomycin. The key parameters of mitochondrial respiration including basal respiration, proton leak, and maximal respiration were calculated within the Seahorse Wave software. The OCR readings were normalized to the cell numbers in each well. Fatty Acid Oxidation (FAO) was measured with a FAO assay kit from Assay Genie (Cat #: BR00001, Dublin, Ireland).

Untargeted lipidomic analysis using LC-MS/MS

To each cell sample, 200 µL of extraction solution containing 2% Avanti SPLASH® LIPIDOMIX® Mass Spec Standard, 1% 10mM butylated hydroxytoluene in ethanol was added and the tubes were vortexed 10 min. The tubes were sat in ice for 10 min and centrifuged at 13,300 rpm for 10 min at 4C. The supernatant was transferred to a glass autosampler vial and

injection volume was 10 µL. Mobile phase A (MPA) was 40:60 acetonitrile: water with 0.1 % formic acid and 10 mM ammonium formate. Mobile phase B (MPB) was 90:9:1 isopropanol: acetonitrile : water with 0.1 % formic acid and 10 mM ammonium formate. The chromatographic method included a Thermo Fisher Scientific Accucore C30 column (2.6 µm, 150 x 2.1 mm) maintained at 40 °C, autosampler tray chilling at 8 °C, a mobile phase flow rate of 0.200 mL/min, and a gradient elution program as follows: 0-3 min, 30% MPB; 3-13 min, 30-43% MPB; 13.1-33 min, 50-70% MPB; 48-55 min, 99% MPB; 55.1-60 min, 30% MPB.

A Thermo Fisher Scientific Orbitrap Fusion Lumos Tribrid mass spectrometer with heated electrospray ionization source was operated in data dependent acquisition mode, in both positive and negative ionization modes, with scan ranges of 150 – 827 and 825 – 1500 *m/z*. An Orbitrap resolution of 120,000 (FWHM) was used for MS1 acquisition and a spray voltage of 3,600 and -2900 V were used for positive and negative ionization modes, respectively. Vaporizer and ion transfer tube temperatures were set at 275 and 300 °C, respectively. The sheath, auxiliary and sweep gas pressures were 35, 10, and 0 (arbitrary units), respectively. For MS^2 and MS^3 fragmentation a hybridized HCD/CID approach was used. Each sample was analyzed using four injections making use of the two aforementioned scan ranges, in both ionization modes.

Lipid data were processed and annotated using Thermo Scientific LipidSearch software (version 5.0) and analyzed using R scripts written in house.

Quantification of Free Fatty Acids (FFA)

Free fatty acids (FFAs) profiling was performed at the Metabolomics Core at MD Anderson Cancer Center, using a chemical derivatization approach. An internal standard mixture consisted of 12.5 µg/mL of (1, 2, 3, 4, 5, 6-$^{13}C_6$) 22:0 and 50 µg/mL ^{13}C-labeled 14:0, 16:1n7c, 16:0, 17:0, 18:2n6, 18:1n9c and 18:0 in ethanol (Cambridge Isotope Laboratories, Tewksbury, MA, USA). To each cell sample in glass tube (prewashed with methanol), 32 µL of internal standard mixture, 1 µL of 10 mM butylated hydroxytoluene in methanol and 1.5 mL of ice-cold methanol were added. The tubes were vortexed for 10 min and sat on iced for 10 min. Following centrifugation at 3,500 *rpm* at 4°C for 10 min, the supernatants were transferred to 2 mL vials with Teflon caps and dried under nitrogen. Extracted FFAs were converted to acyl chloride intermediates by treatment with 300 µL of 2M oxalyl chloride in dichloromethane at 65°C for 5 min. The solutions were then dried and samples were derivatized by adding 225 µL of 1% (v/v) 3-picolylamine in acetonitrile. After sitting in room temperature for 5 min, the solutions were dried and derivatization products were reconstituted in 100 µL ethanol. Injection volume was 10 µL. Mobile phase A (MPA) was 0.1% formic acid in water, and mobile phase B (MPB) was 0.1% formic acid in acetonitrile. The chromatographic method included a Thermo Fisher Scientific Accucore C30 column (2.6 µm, 150 x 2.1mm) and the following gradient elution: 0-5 min, 5% MPB; 5-45 min, 95% MPB; 45-85 min, 95% MPB; 85.1-90 min, 5% MPB. Flow rate was set at 0.5 mL/min. A Thermo Fisher Scientific Orbitrap Fusion Tribrid mass spectrometer with heated electrospray ionization source was operated in data dependent acquisition mode with a scan range of 150-550*m/z*. Orbitrap resolutions of 120,000 (FWHM) and 30,000 for MS1 and MS2 were used, respectively. The instrument was operated in positive ionization mode with a spray voltage of 3,600 V, and vaporizer and capillary temperatures set at 350 and 325 °C, respectively. The sheath, auxiliary and sweep gas pressures were 50, 10, and 1 (arbitrary units), respectively. Ions were fragmented using assisted HCD with stepped collision energies of 25, 30, and 35%.

Western blotting

Protein samples were lysed in NENT buffer (100 mM NaCl, 20 mM Tris-Cl (pH 8.0), 0.5 mM EDTA, 0.5% (v/v) NP-40) with protease inhibitors and DTT, except that the normal human liver lysates were directly ordered from Santa Cruz Biotechnology (Santa Cruz, California). The samples were then spun at 12,000 x g for 10 minutes at 4 °C and the supernatant was collected and subsequently heated for the protein SDS PAGE analysis. Specifically, for the SCD proteins, the samples were incubated at room temperature for 10 minutes upon mixing with SDS loading buffer (without boiling). Protein samples were separated by the mini SDS gel and transferred to polyvinylidene difluoride (PVDF) membranes where they were then blocked in 5% fat free milk. Afterward, the PVDF membranes were incubated with primary antibodies at 4°C overnight or room temperature (RT) for 3 hours and then washed with PBST (0.1% Tween 20 in 1 x PBS, pH = 7.4) for 3 times (5 min/each wash). Following this, the membranes were incubated with IRDye 800 CW or 680 RD secondary antibodies (LI-COR) at RT for 1 hour. After being washed with PBST 3 times (5 min/each wash), the blots were imaged by Odyssey software (LI-COR Biosciences) and the band densities were analyzed by ImageJ software. For the Western blotting, the following primary antibodies were used: CES1 (Invitrogen, MA5-24244), PLIN2 (abcam, ab52356), PLIN3 (Abcam, ab47639), PLIN5 (Abcam, ab228111), CIDEA (Abcam, ab8402), CIDEC (Abcam, ab198204), BIP (ThermoFisher Scientific, PA1–014A), ATGL (Santa Cruz Biotechnology, sc-36527), CGI58 (Santa Cruz Biotechnology, sc-365278), HSL (Cell Signaling Technology, 4107), pHSL (Ser660) (Cell Signaling Technology, 4126), SCD1 (Cell Signaling Technology, 2438), ACC1 (Cell Signaling Technology, 4190), pACC1 (Cell Signaling Technology, 3661), FASN (Cell Signaling Technology, 3180), DGAT2 (Thermofisher Scientific, PA5-103785), OXPHOs Human WB antibody Cocktail (Invitrogen, 45-8199), BiP (ThermoFisher Scientific, PA1–014A), XBP (Santa Cruz Biotechnology, sc-271878), AKT (Cell Signaling Technology, 4691), p-AKT (Cell Signaling Technology, 3038), Caspase-3 (Cell Signaling Technology, 9664), β-ACTIN (BD Biosciences, 61256), α-Tubulin (Cell Signaling Technology, 2144).

Quantitative PCR (qPCR)

Total RNAs from HepG2 cells were isolated using Trizol reagent (Thermo Fisher Scientific, #15596018) following the manufacturer's instructions. For qPCR, cDNAs were obtained by reverse-transcribing 1 μg of total RNAs with RevertAid Reverse Transcription Kit (Thermo Fisher Scientific, #K1691). qPCR reactions were carried out on Bio-Rad CFX96 system (Bio-Rad Laboratories). Results were normalized by β-actin and calculated using the 2-ΔΔCt method. The primers were listed in Supplementary Table 1. Each biological replicate was multiple-well analysis at the same time. Meanwhile, the related experiments were repeated for three times.

Fluorescent labeling of lipid droplets and immunofluorescence (IF) staining of the cells

To label the lipid droplets in cells, 5 μg/ml of BODIPY 493/503 (Thermo Fisher Scientific, #D3922) was added to the cell culture media and incubated for 1 hour. The cells were then washed with warm 1 x PBS 3 times and fixed by 4% paraformaldehyde for 1 h. They were then imaged using a Leica TCS SP5 Confocal Laser Scanning Microscope. For immunofluorescence staining, the cells were washed with PBS three times and fixed in 4% paraformaldehyde for 30 min. After being washed by 1 x PBST for 3 times (5 min/wash) and blocked in 5% bovine serum albumin for 1 h, the cells were then stained with indicated primary antibodies at 4°C for overnight. The cells were then washed with 1 x PBST for 3 times (5 min/wash) and incubated with indicated secondary antibodies at RT for 1 h. After being washed with 1 x PBST for 3 times (5 min/wash), the cells were mounted and images were acquired with a Leica TCS SP5 Confocal Laser Scanning Microscope.

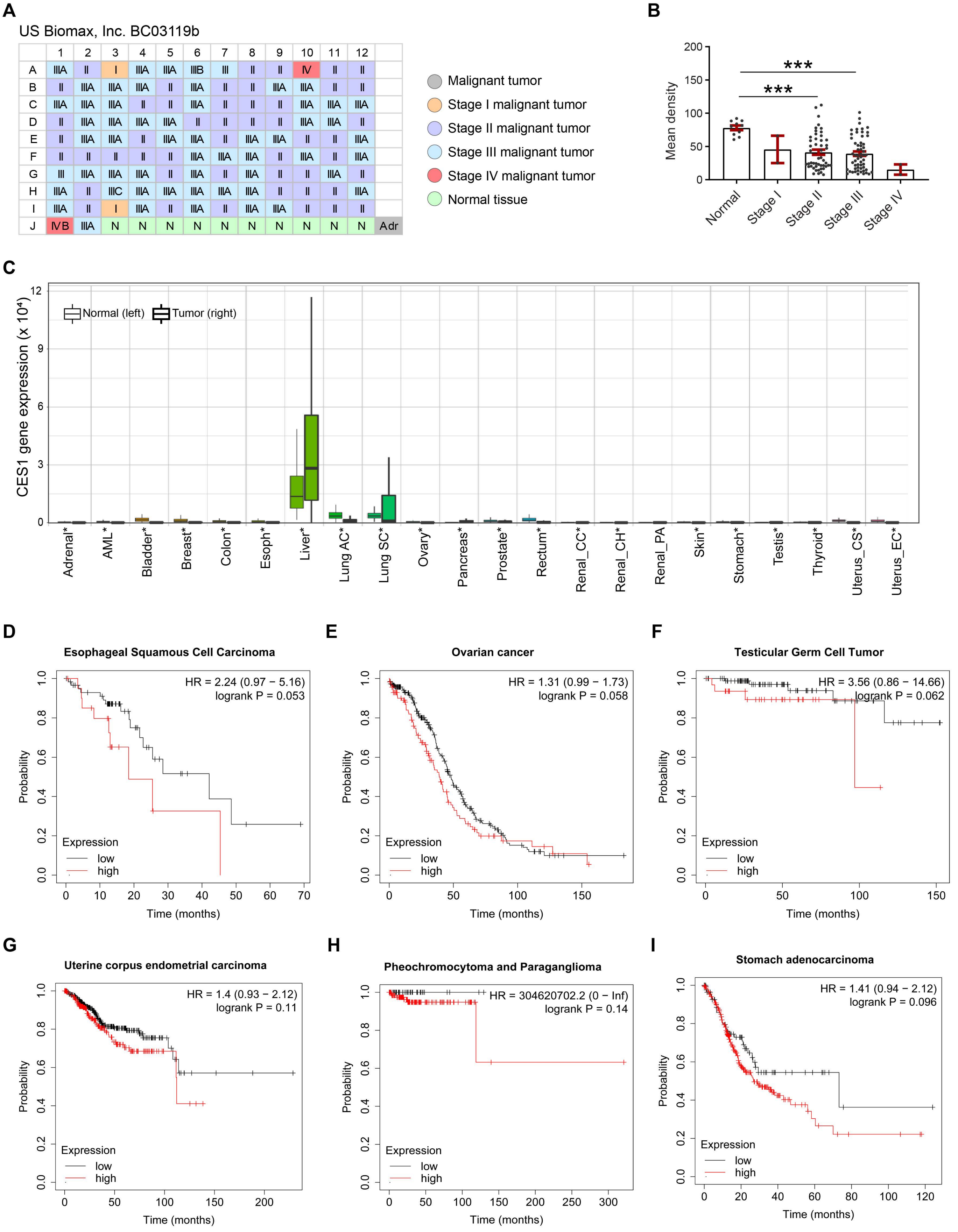

	1	2	3	4	5	6	7	8	9	10	11	12	
A	IIIA	II	I	IIIA	IIIA	IIIB	III	II	II	IV	II	II	
B	II	IIIA	IIIA	IIIA	II	IIIA	II	II	IIIA	IIIA	II	II	
C	IIIA	IIIA	IIIA	II	II	IIIA	II	II	II	IIIA	IIIA	IIIA	
D	II	IIIA	IIIA	IIIA	IIIA	II	II	II	II	IIIA	IIIA	II	
E	II	IIIA	IIIA	II	IIIA	IIIA	II	IIIA	IIIA	II	II	IIIA	
F	II	II	II	II	II	IIIA	IIIA	IIIA	II	IIIA	II	IIIA	
G	III	IIIA	IIIA	IIIA	II	IIIA	II	IIIA	II	II	IIIA	II	
H	IIIA	II	IIIC	IIIA	IIIA	IIIA	IIIA	IIIA	II	II	II	IIIA	
I	IIIA	II	I	IIIA	II	IIIA	II	IIIA	IIIA	II	II	II	
J	IVB	IIIA	N	N	N	N	N	N	N	N	N	N	Adr

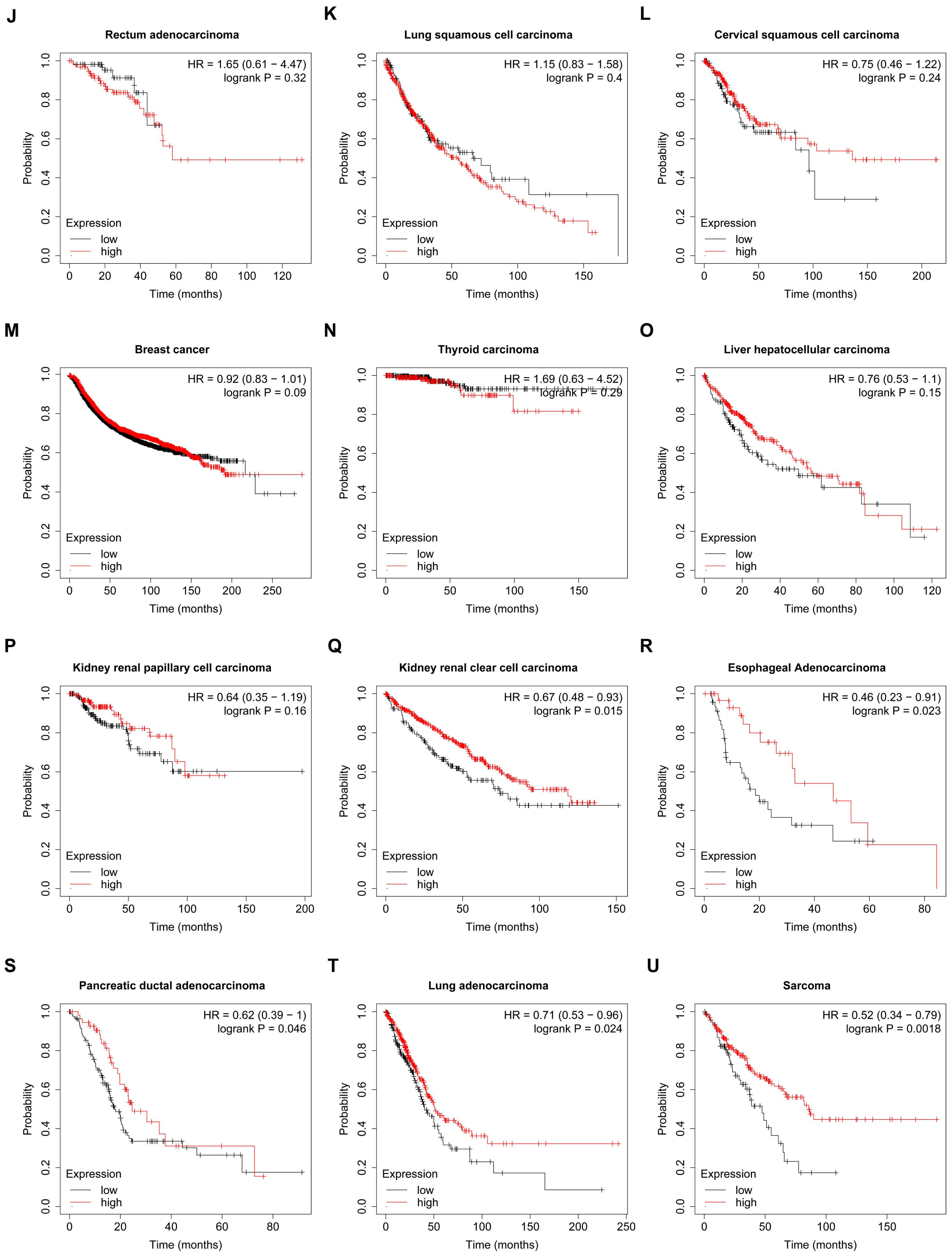
J
Rectum adenocarcinoma
HR = 1.65 (0.61 − 4.47)
logrank P = 0.32
K
Lung squamous cell carcinoma
HR = 1.15 (0.83 − 1.58)
logrank P = 0.4
L
Cervical squamous cell carcinoma
HR = 0.75 (0.46 − 1.22)
logrank P = 0.24
M
Breast cancer
HR = 0.92 (0.83 − 1.01)
logrank P = 0.09
N
Thyroid carcinoma
HR = 1.69 (0.63 − 4.52)
logrank P = 0.29
O
Liver hepatocellular carcinoma
HR = 0.76 (0.53 − 1.1)
logrank P = 0.15
P
Kidney renal papillary cell carcinoma
HR = 0.64 (0.35 − 1.19)
logrank P = 0.16
Q
Kidney renal clear cell carcinoma
HR = 0.67 (0.48 − 0.93)
logrank P = 0.015
R
Esophageal Adenocarcinoma
HR = 0.46 (0.23 − 0.91)
logrank P = 0.023
S
Pancreatic ductal adenocarcinoma
HR = 0.62 (0.39 − 1)
logrank P = 0.046
T
Lung adenocarcinoma
HR = 0.71 (0.53 − 0.96)
logrank P = 0.024
U
Sarcoma
HR = 0.52 (0.34 − 0.79)
logrank P = 0.0018
Probability
Time (months)
Expression
low
high

Supplementary Figure 1. CES1 is selectively expressed in HCC and is associated with survival in different cancer patients.

A, The sample information about the human liver tissue array used in Figure 1A.

B, Analysis of the fluorescence intensity of CES1 staining in Figure 1A at different tumor development stages (stage I to IV).

C, Comparison of CES1 expression in normal and tumor tissues. Results was acquired from database in the TNMplot (tnmplot.com/analysis/). Significant differences were analyzed by a Mann-Whitney U test. * $p < 0.05$.

D-U, Correlation analysis of CES1 expression and survival rate in different cancer types. Data was generated using KM plotter (kmplot.com).

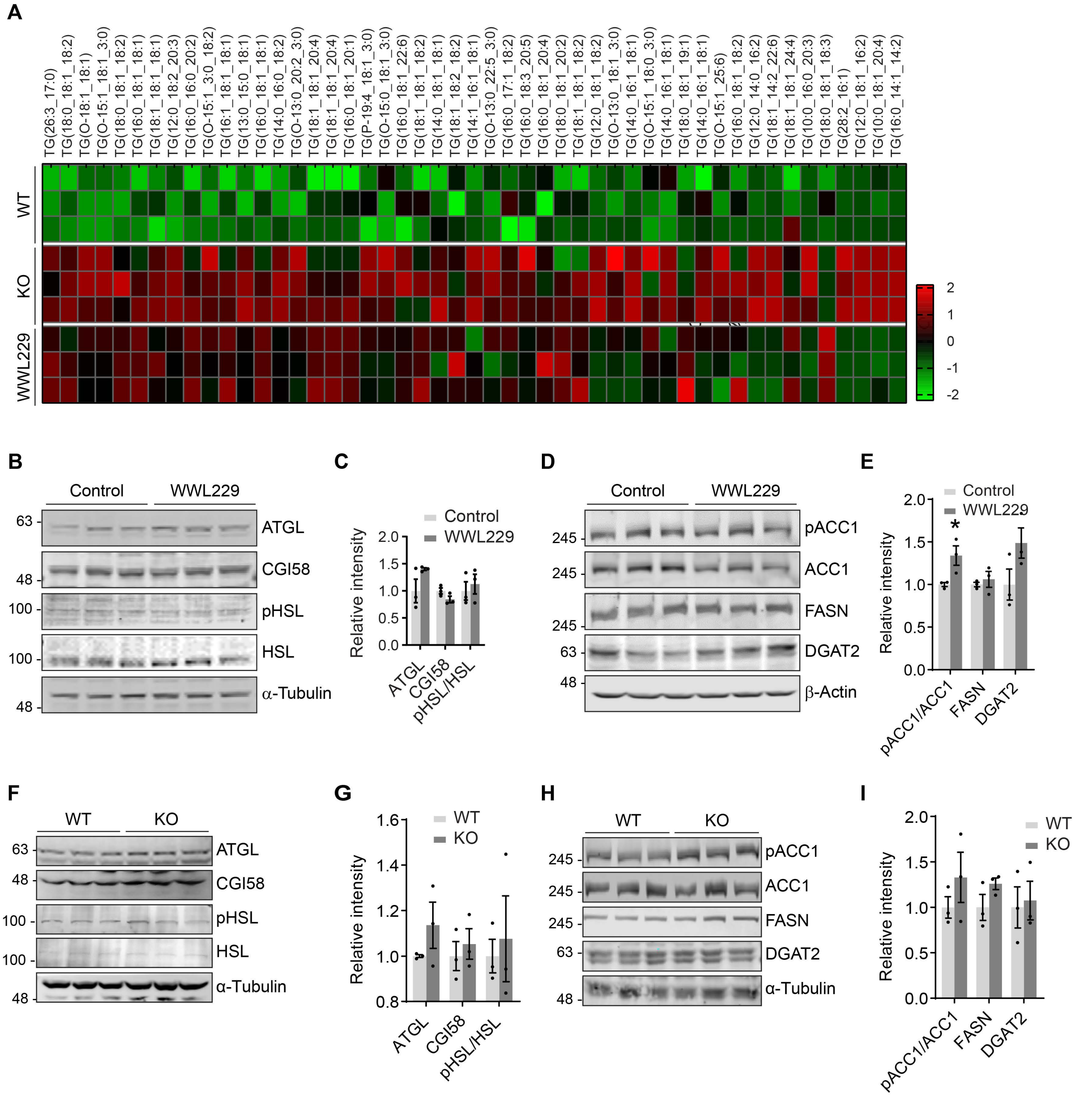
A
WT
KO
WWL229
2
1
0
-1
-2
B
Control
WWL229
ATGL
CGI58
pHSL
HSL
α-Tubulin
C
Control
WWL229
Relative intensity
ATGL
CGI58
pHSL/HSL
D
pACC1
ACC1
FASN
DGAT2
β-Actin
E
pACC1/ACC1
FASN
DGAT2
F
WT
KO
G
H
I

Supplementary Figure 2. CES1 affects lipid droplets morphology and lipidomic profiles.

A, Heatmap showing the top 50 TG levels in wild-type (WT), CES1 KO (KO), and WWL229 treated HepG2 cells. For WWL229 treatment, the cells were treated with 50 μM WWL229 for 24 h. The data are shown as log2 fold changes (FC, log2-transformed, and scaled accordingly).

B, WB analysis of ATGL, CGI58, pHSL, and total HSL in lysates from HepG2 cells treated with or without 50 μM WWL229 for 48 h. α-Tubulin was used as a loading control (n = 3 per group. Representative of three repeats).

C, Quantification of the band intensity in (B) (n = 3 per group, each point represents a biological replicate. Data are presented as mean ± SEM, *Student's t-test*).

D, WB analysis of pACC1, ACC1, FASN, and DGAT2 in lysates from HepG2 cells treated with or without 50 μM WWL229 for 48 h. β-actin was used as the loading control (n = 3 per group. Representative of three repeats).

E, Quantification of the band intensity in (D) (n = 3 per group, each point represents a biological replicate. Data are presented as mean ± SEM, *Student's t-test*, * $p < 0.05$).

F, WB analysis of ATGL, CGI58, pHSL, and HSL in lysates from wild-type (WT) and CES1 KO (KO) HepG2 cells. α-Tubulin was used as the loading control (n = 3 per group. Representative of three repeats).

G, Quantification of the band intensity in (F) (n = 3 per group, each point represents a biological replicate. Data are presented as mean ± SEM, *Student's t-test*).

H, WB analysis of pACC1, ACC1, FASN, and DGAT2 in lysates from WT and KO HepG2 cells. α-Tubulin was used as the loading control (n = 3 per group. Representative of three repeats).

I, Quantification of the band intensity in (H) (n = 3 per group, each point represents a biological replicate. Data are presented as mean ± SEM, *Student's t-test*).

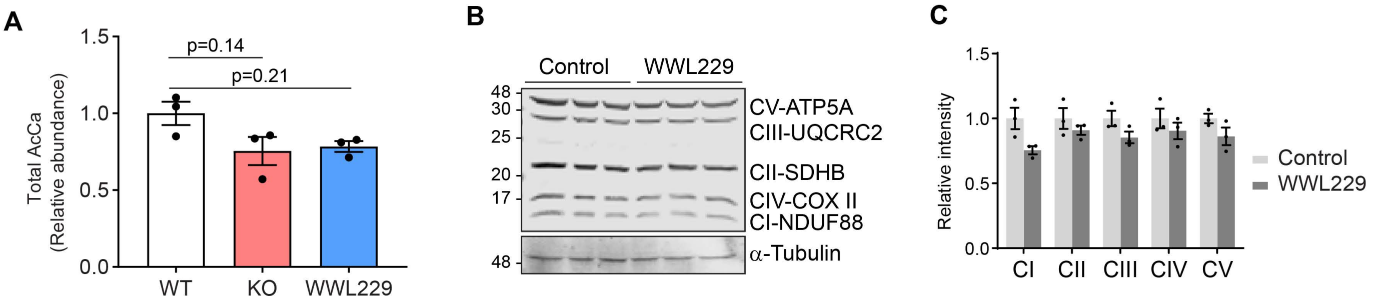

Supplementary Figure 3. Blockage of CES1 impairs mitochondrial function.

A, Comparison of the total acylcarnitine levels in the WT, KO, and WWL229 treated HepG2 cells (n = 3 per group. Data are represented as mean ± SEM, One-way ANOVA followed by Dunnett T3-test).

B, WB analysis of OXPHOS complexes subunits I-V (CI-CV) in the lysates from the HepG2 cells treated with or without 50 μM WWL229 for 48 hours. The antibodies of NDUF88, SDHB, UQCRC2, COX II, and ATP5A were used to label CI-CV, respectively. α-Tubulin was used as loading control (n = 3 per group. Representative of three repeats).

C, Quantification of the band intensity in (A) (n = 3 per group, each point represents a biology replicate. Data are represented as mean ± SEM, *Student's t-test*).

Supplementary Figure 4 **Li., *et al.* 2022**

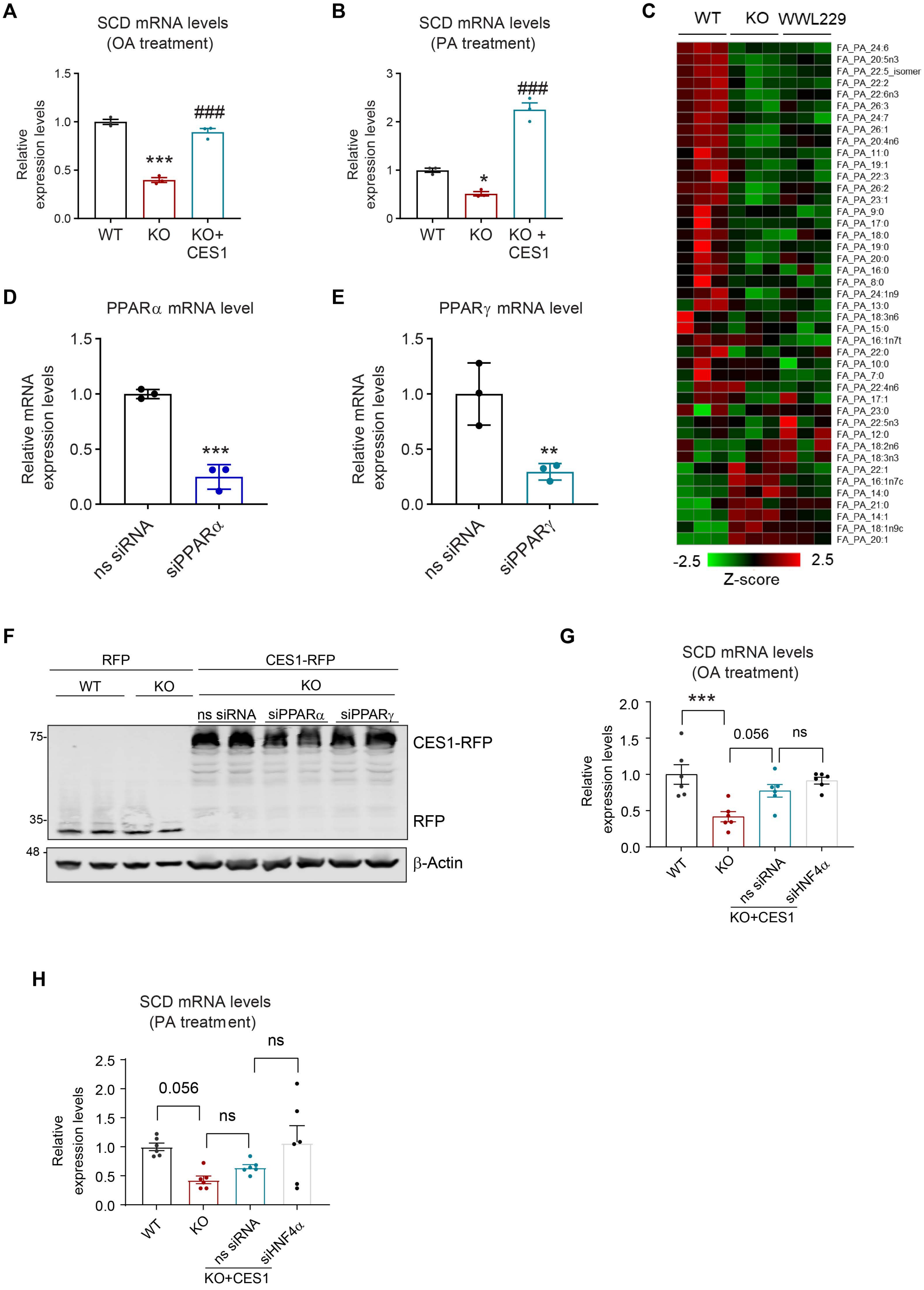

Supplementary Figure 4. Blockage of CES1 activity decreases SCD levels through PPARα/γ

A, qPCR analysis of SCD levels in the WT, KO, KO with re-expression of CES1 (KO + CES1) HepG2 cells treated with 200 μM OA for 12 hours (n = 3 per group, each point represents a biology replicate. Data are represented as mean ± SEM, One-way ANOVA followed by Dunnett T3-test, *** $p < 0.001$ vs. WT, ### $p < 0.001$ vs. KO).

B, qPCR analysis of SCD levels in the WT, KO, KO with re-expression of CES1 (KO + CES1) HepG2 cells treated with 100 μM PA for 12 hours (n = 3 per group, each point represents a biology replicate. Data are represented as mean ± SEM, One-way ANOVA followed by Dunnett T3-test, * $p < 0.05$ vs. WT, ### $p < 0.001$ vs. KO).

C, Heatmap showing the difference of free fatty acids in WT, KO, and WWL229 treated HepG2 cells.

D, qPCR analysis of PPARα levels in the KO cells transfected with ns siRNA or siPPARα (n = 3 per group, each point represents a biology replicate. Data are represented as mean ± SD, *Student's t-test,* ***$p<0.001$)

E, qPCR analysis of PPARγ levels in the KO cells transfected with ns siRNA or siPPARγ (n = 3 per group, each point represents a biology replicate. Data are represented as mean ± SD, *Student's t-test,* **$p<0.01$).

F, WB analysis of CES1 levels in the lysates from HepG2 WT, KO, KO with re-expression of CES1-RFP (KO + CES1), and re-expressed CES1 KO cells transfected with ns siRNA, siPPARα, or siPPARγ (n = 4 per group. Representative of two repeats).

G, qPCR analysis of SCD levels in the WT, KO, KO with re-expression of CES1 (KO + CES1), and re-expressed CES1 KO cells transfected with siHNF4α (KO + CES1 + siHNF4α). The cells were treated with 200 μM OA for 12 hours (n = 3 per group, each point represents a biology replicate. Data are represented as mean ± SEM, One-way ANOVA followed by Dunnett T3-test, *** $p < 0.001$).

H, qPCR analysis of SCD levels in the WT, KO, KO with re-expression of CES1 (KO + CES1), and re-expressed CES1 KO cells transfected with siHNF4α(KO + CES1 + siHNF4α). The cells were treated with 100 μM PA for 12 hours (n = 3 per group, each point represents a biology replicate. Data are reprevsented as mean ± SEM, One-way ANOVA followed by Dunnett T3-test).

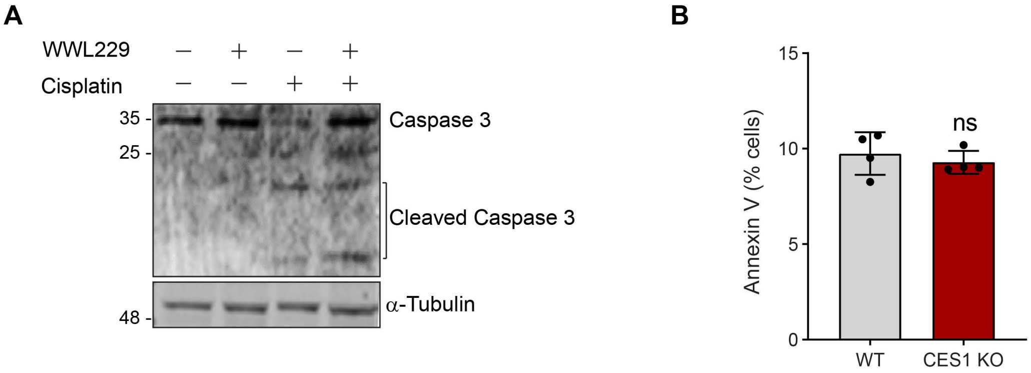

Supplementary Figure 5. Blockage of CES1 activity sensitizes HCC for chemotherapy.

A, WB analysis of full-length and cleaved caspase 3 levels in WT HepG2 cells treated by 50 μM WWL229 together with or without 10 μM cisplatin for 48 hours (n = 3 per group. Representative of three repeats). The cleaved bands indicate more apoptotic cells in the samples.

B, Cell apoptosis assay of WT and KO HepG2 cells. Apoptotic cells were detected using annexin V staining and quantified using flow cytometry (n = 4 per group, each point represents a biological replicate. Data are represented as mean ± SD, *Student's t-test*).

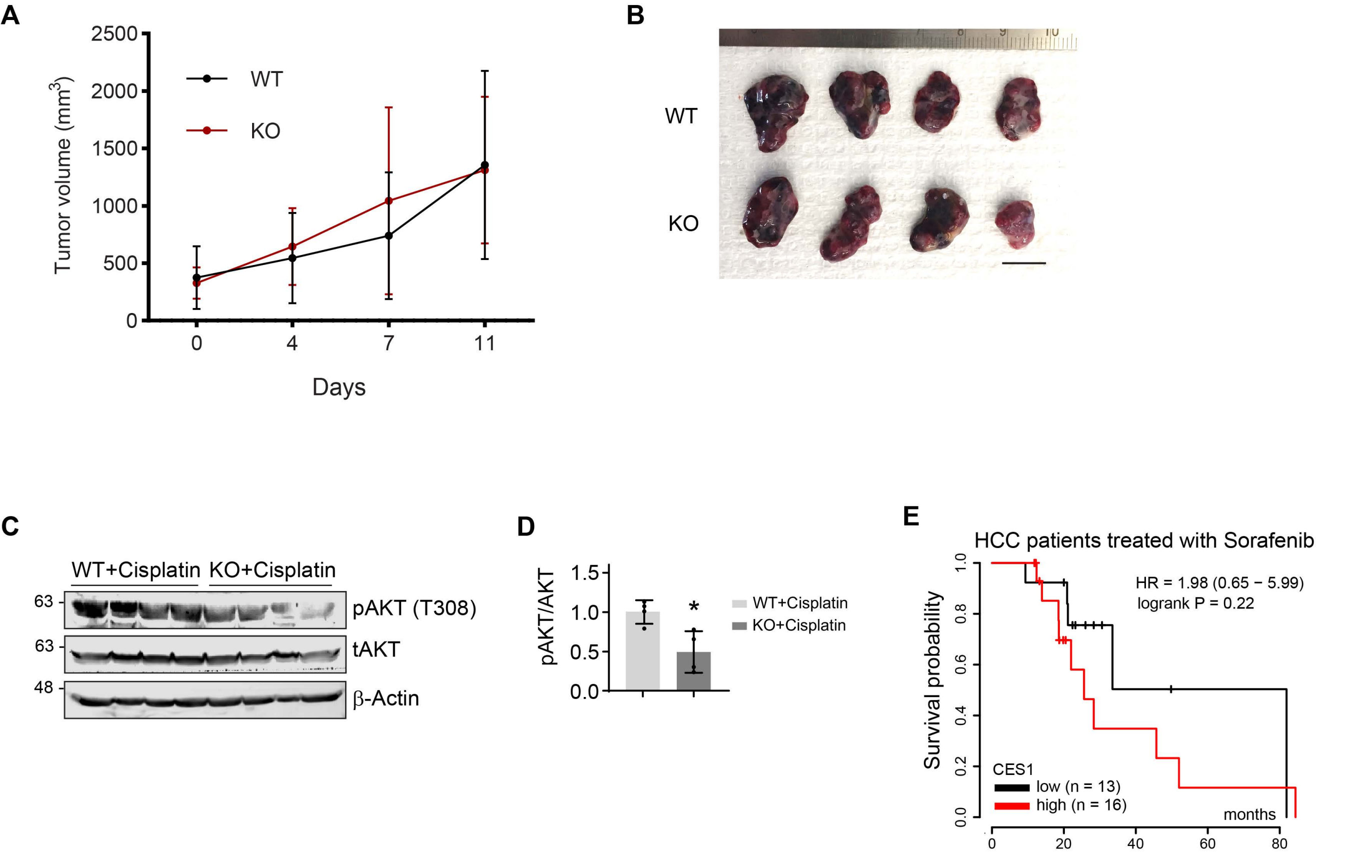

Supplementary Figure 6. Blockage of CES1 activity significantly enhances cisplatin inhibition of tumor growth in HepG2 xenografted NU/J mice.

A, Measurement of tumor growth of xenografts formed by injected HepG2 WT and CES1 KO cells in NU/J mice (n = 4 per group, data are presented as mean ± SD, Two-way ANOVA followed by Tukey multiple comparisons test).

B, Images of xenograft biopsies collected from mice in (A) after 11 day of measurement. Scal bar = 1 cm.

C, WB analysis of pAKT (T308) and total AKT in the lysates from the WT and KO HepG2 xenografts after 16 days of cisplatin treatment. β-Actin was used as loading control (n = 3 per group. Representative of three repeats).

D, Quantification of the band intensity in (C) (n = 3 per group, each point represents a biology replicate. Data are represented as mean ± SEM, *Student's t-test*, * $p < 0.05$).

E, Correlation analysis of CES1 expression and survival rate in sorafenib-treated HCC patients. The data were generated using a KM plotter (kmplot.com).

Supplementary Table 1

Primers Used for the qPCRs

**Gene name**	**Forward (5' to 3')**	**Reverse (5' to 3')**
ACADS	AGCGGCTCAGGTGAAGAAG	GCGTAGGCCAGGTAATCGAG
ACADM	TGGATAACCAACGGAGGAAAAG	CTGGGGTATCTGCTTCCACA
ADADVL	TAGGAGAGGCAGGCAAACAGCT	CACAGTGGCAAACTGCTCCAGA
CPT1A	GATCCTGGACAATACCTCGGAG	CTCCACAGCATCAAGAGACTGC
CPT2	GCAGATGATGGTTGAGTGCTCC	AGATGCCGCAGAGCAAACAAGTG
ECH1	CGATACCAGGAGACCTTCAACG	GGAAGAAAGCATCCTGGGCACA
PGC1a	GGAACTGCAGGCCTAACTCC	CACTGTCCCTCAGTTCACCG
TFAM1	ATGCTTATAGGGCGGAGTGG	TGGTTTCCTGTGCCTATCCA
NRF1	CCAGTGGCCACACAGAACTC	CTTCCTTTCCCTTCCACTGC
SCD	TCTTCTCTCACGTGGGTTGG	AGCCAGGTTTGTAGTACCTCCT
SOD1	AGGGCATCATCAATTTCGAG	CCATCTTTGTCAGCAGTCAC
SOD2	AGAAGTACCAGGAGGVGTTG	AGTGTCCCCGTTCCTTATTG
GPX1	GAAGTGCGAGGTGAACGGTG	GGGATCAACAGGACCAGCAC
CAT	CTGGGACTTCTGGAGCCTAC	CAACTGGGATGAGAGGGTAG
BIP	TGTTCAACCAATTATCAGCAAACTC	TTCTGCTGTATCCTCTTCACCAGT
XBP1s	CTGAGTCCGAATCAGGTGCAG	ATCCATGGGGAGATGTTCTGG
XBP1u	CAGCACTCAGACTACGTGCA	ATCCATGGGGAGATGTTCTGG
