# Supplementary material for: Interfering with lipid metabolism through targeting CES1 sensitizes hepatocellular carcinoma for chemotherapy
Source: JCI Insight. 2023 Jan 24;8(2):e163624. doi: 10.1172/jci.insight.163624 (PMC9977307; doi:10.1172/jci.insight.163624)
Supplement: Supplemental table 1 [file jciinsight-8-163624-s136.pdf]

Supplementary Table 1

Primers Used for the qPCRs

**Gene name**	**Forward (5' to 3')**	**Reverse (5' to 3')**
ACADS	AGCGGCTCAGGTGAAGAAG	GCGTAGGCCAGGTAATCGAG
ACADM	TGGATAACCAACGGAGGAAAAG	CTGGGGTATCTGCTTCCACA
ADADVL	TAGGAGAGGCAGGCAAACAGCT	CACAGTGGCAAACTGCTCCAGA
CPT1A	GATCCTGGACAATACCTCGGAG	CTCCACAGCATCAAGAGACTGC
CPT2	GCAGATGATGGTTGAGTGCTCC	AGATGCCGCAGAGCAAACAAGTG
ECH1	CGATACCAGGAGACCTTCAACG	GGAAGAAAGCATCCTGGGCACA
PGC1a	GGAACTGCAGGCCTAACTCC	CACTGTCCCTCAGTTCACCG
TFAM1	ATGCTTATAGGGCGGAGTGG	TGGTTTCCTGTGCCTATCCA
NRF1	CCAGTGGCCACACAGAACTC	CTTCCTTTCCCTTCCACTGC
SCD	TCTTCTCTCACGTGGGTTGG	AGCCAGGTTTGTAGTACCTCCT
SOD1	AGGGCATCATCAATTTCGAG	CCATCTTTGTCAGCAGTCAC
SOD2	AGAAGTACCAGGAGGVGTTG	AGTGTCCCCGTTCCTTATTG
GPX1	GAAGTGCGAGGTGAACGGTG	GGGATCAACAGGACCAGCAC
CAT	CTGGGACTTCTGGAGCCTAC	CAACTGGGATGAGAGGGTAG
BIP	TGTTCAACCAATTATCAGCAAACTC	TTCTGCTGTATCCTCTTCACCAGT
XBP1s	CTGAGTCCGAATCAGGTGCAG	ATCCATGGGGAGATGTTCTGG
XBP1u	CAGCACTCAGACTACGTGCA	ATCCATGGGGAGATGTTCTGG
